# An Update on *Candida tropicalis* Based on Basic and Clinical Approaches

**DOI:** 10.3389/fmicb.2017.01927

**Published:** 2017-10-13

**Authors:** Diana L. Zuza-Alves, Walicyranison P. Silva-Rocha, Guilherme M. Chaves

**Affiliations:** Laboratory of Medical and Molecular Mycology, Department of Clinical and Toxicological Analyses, Federal University of Rio Grande do Norte, Natal, Brazil

**Keywords:** *Candida tropicalis*, virulence factors, antifungal resistance, phenotypic and molecular identification, update

## Abstract

*Candida tropicalis* has emerged as one of the most important *Candida* species. It has been widely considered the second most virulent *Candida* species, only preceded by *C. albicans*. Besides, this species has been recognized as a very strong biofilm producer, surpassing *C. albicans* in most of the studies. In addition, it produces a wide range of other virulence factors, including: adhesion to buccal epithelial and endothelial cells; the secretion of lytic enzymes, such as proteinases, phospholipases, and hemolysins, bud-to-hyphae transition (also called morphogenesis) and the phenomenon called phenotypic switching. This is a species very closely related to *C. albicans* and has been easily identified with both phenotypic and molecular methods. In addition, no cryptic sibling species were yet described in the literature, what is contradictory to some other medically important *Candida* species. *C. tropicalis* is a clinically relevant species and may be the second or third etiological agent of candidemia, specifically in Latin American countries and Asia. Antifungal resistance to the azoles, polyenes, and echinocandins has already been described. Apart from all these characteristics, *C. tropicalis* has been considered an osmotolerant microorganism and this ability to survive to high salt concentration may be important for fungal persistence in saline environments. This physiological characteristic makes this species suitable for use in biotechnology processes. Here we describe an update of *C. tropicalis*, focusing on all these previously mentioned subjects.

## Introduction

In the last decades, medicine advances related to the discovery of several medical devices which seek for a longer survival of patients with several infirmities, such as AIDS, hematological malignancies, cancer, and other immunosuppressive diseases promoted a longer lifespan. On the other hand, the number of opportunistic fungal infections increased, mainly the ones caused by the *Candida* genus (Pincus et al., 2007; Araújo et al., 2017). In this context, *Candida tropicalis* emerges as one of the most important *Candida* species in terms of epidemiology and virulence. It is able to produce true hyphae, an exclusive property of *Candida albicans* and its sibling species *Candida dubliniensis. C. tropicalis* has also been considered a strong biofilm producer species and is highly adherent to epithelial and endothelial cells (Marcos-Zambrano et al., 2014). In addition, several recent investigations have reported the recovery of *C. tropicalis* resistant to the antifungal drugs currently available, such as the azoles derivatives, amphotericin B, and echinocandins (Choi et al., 2016; Seneviratne et al., 2016). In addition, *C. tropicalis* has been considered an osmotolerant microorganism and this ability to survive to high salt concentration may be important for fungal persistence in saline environments, contributing to the expression of virulence factors *in vitro* and resistance to antifungal drugs (Zuza-Alves et al., 2016). This property explains *C. tropicalis* potential use in biotechnological processes such as the production of xylitol from corn fiber and the ethanol from marine algae (Rao et al., 2006; Ra et al., 2015).

## Biology and taxonomy

*C. tropicalis* was originally isolated from a patient with fungal bronchitis in 1910 and named *Oidium tropicale* (Castellani, 1912). It is a yeast belonging to the filo Ascomycota, from the Hemiascomycetes class (Blandin et al., 2000), which has a single Order created in 1960 by Kudrjavzev, called Saccharomycetales (Kirk et al., 2001). This monophyletic lineage comprises about 1,000 known species, including several yeasts of medical importance such as *C. tropicalis* (Diezmann et al., 2004).

**Figure 1:**
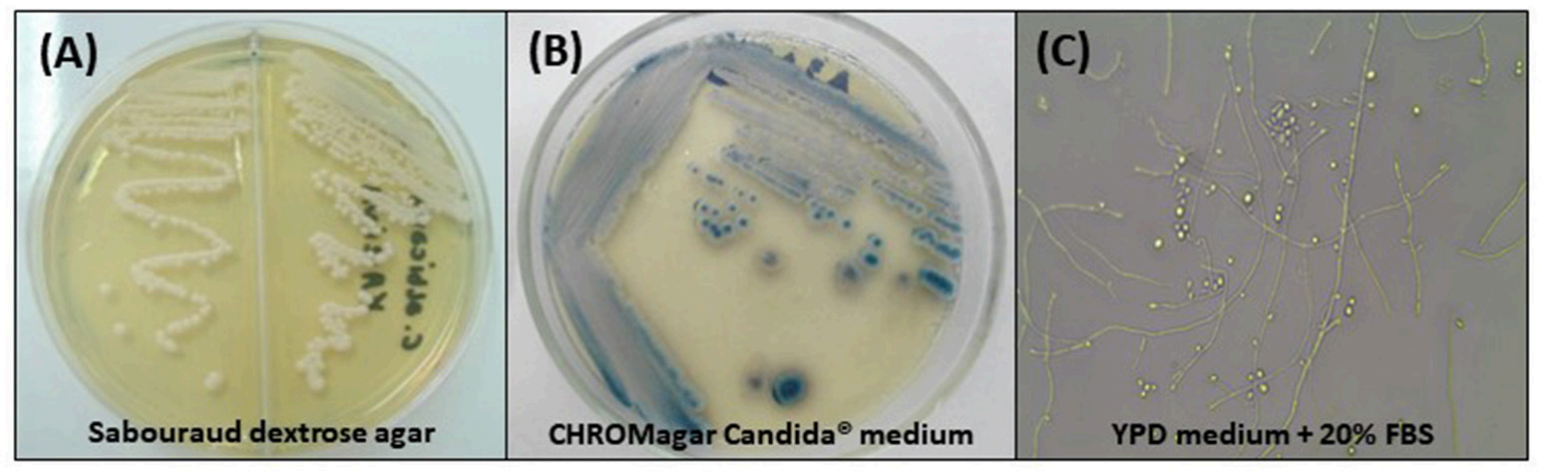
Phenotypic characteristics of *Candida tropicalis*. **(A)** Cream-colored, dull, smooth colonies, after 48 h of incubation at 30°C on Sabouraud dextrose agar; **(B)** Colonies with typical dark blue color on CHROMagar Candida® medium after 96 h of incubation at 35°C; **(C)** Micromorphological aspects after incubation in YPD medium containing 20% fetal bovine serum (FBS) for 7 days at 30°C, 400x: blastoconidia in single or branched chains, true hyphae and abundant pseudohyphae.

According to Kurtzman et al. (2011) *C. tropicais* colonies on Sabouraud Dextrose Agar (SDA) are white to cream, with a creamy texture and smooth appearance and may have slightly wrinkled edges. Therefore, it is indistinguishable from other *Candida* species. After 7 days of microculture on cornmeal agar containing Tween 80, incubated at 25°C, spherical or ovoid blastoconidia, which may be grouped in pairs or alone, measuring ~4–8 × 5–11 μm, pseudohyphae in branched chains, and even true hyphae may be observed (Silva et al., 2012; Figure 1). With respect to the biochemical characteristics, it is known that *C. tropicalis* is capable of fermenting galactose, sucrose, maltose, and trehalose, besides assimilating these and others carbohydrates through the oxidative pathway (Kurtzman et al., 2011).

## Genetic characteristic

*C. tropicalis* is a diploid yeast, whose genome was sequenced in 2009 (strain MYA-3404) in a study conducted by Butler et al. (2009). It has a genomic size of 14.5 Mb, containing 6,258 genes encoding proteins and a guanine-cytosine content of 33.1%. The number of chromosomes is not known with precision, but Doi et al. reported 12 chromosomes per cell for *C. tropicalis* (Doi et al., 1992).

It has been widely believed that *C. tropicalis* is an asexual yeast. However, some studies performed recently have reported that mating between diploid cells a and α, generating a/α tetraploid cells may occur (Porman et al., 2011; Xie et al., 2012; Seervai et al., 2013). Such mating is regulated by colony phenotypic switching, where cells change from a white to an opaque state. Seervai et al. (2013) demonstrated that tetraploid strains of *C. tropicalis* can be induced to undergo parasexual cycle without meiotic reduction. This process results in a or α diploid cells competent for mating, being able to form tetraploid cells, which show chromosomal instability after incubation and return to the diploid state after ~240 generations (Seervai et al., 2013). Genetic recombination has also been demonstrated, besides ploidy changes (aneuploidies and polyploidy), affecting cells gene expression and protein production (Morrow and Fraser, 2013). This reduction in ploidy is considered a mechanism of adaptation and may be associated with cell stress (Berman and Hadany, 2012). This adaptive mechanism may also generate karyotype variation within the host, and may be induced by various stressors, such as thermal shock, exposure to UV light, and growth in l-sorbose or d-arabinose as the only carbon source (Legrand et al., 2008; Arbour et al., 2009; Bouchonville et al., 2009; Morrow and Fraser, 2013). It is important to emphasize again that meiosis occurrence has never been described in *C. tropicalis*.

**Figure 2:**
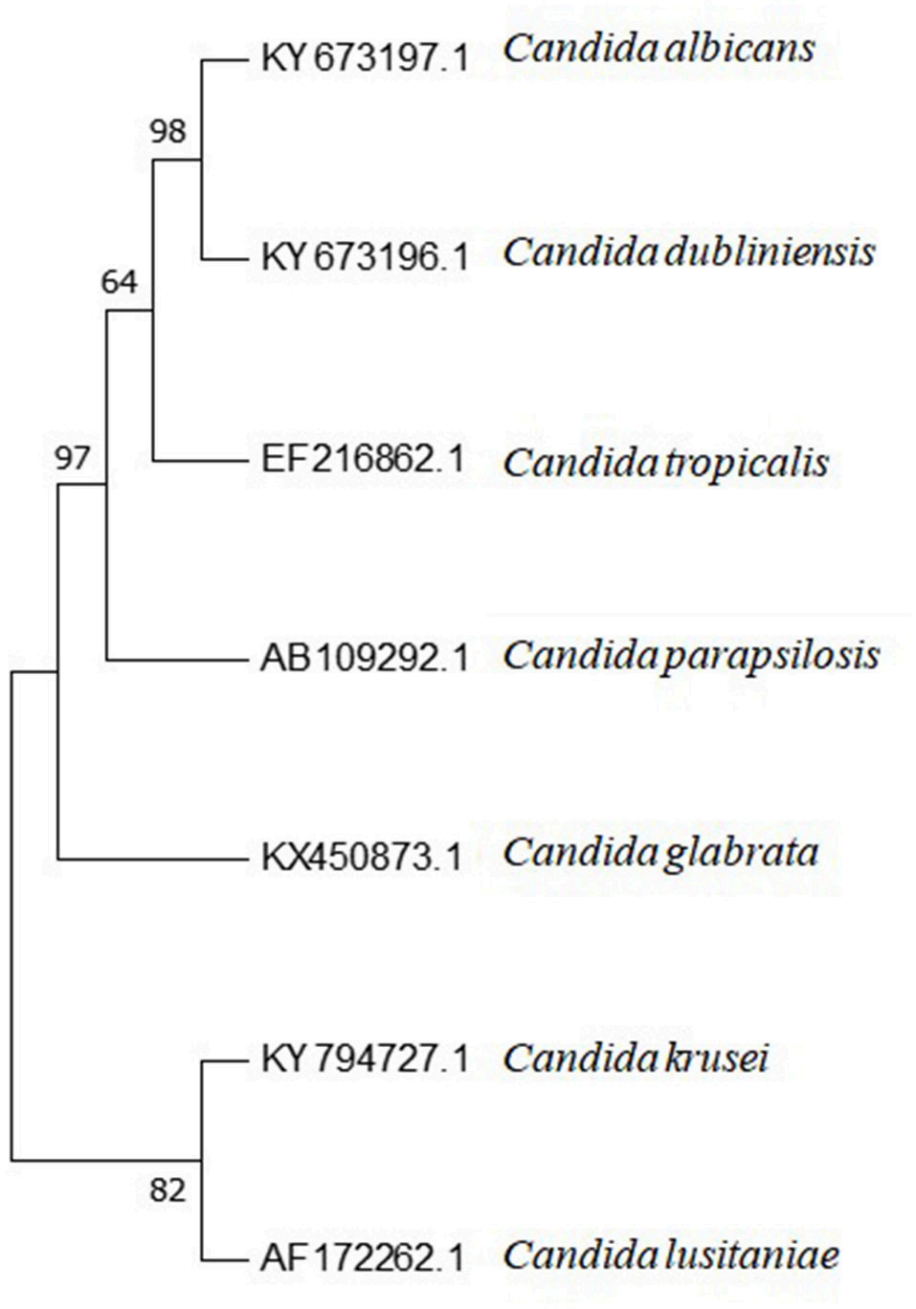
Phylogenetic tree of *Candida* spp. internal transcribed spacer 1 (ITS1)-5.8S ribosomal RNA gene and internal transcribed spacer 2 (ITS2) complete sequences and their accession numbers, obtained from Genbank database at https://www.ncbi.nlm.nih.gov. Sequences were aligned using BioEdit software (v7.2.61). Aligned sequences were used for phylogenetic analysis conducted with Mega 7.0.26 Software. The method used for tree constructions was maximum parsimony. Phylogram stability was accessed by bootstrapping with 1,000 pseudoreplications.

*C. tropicalis* has greater genetic similarity with *C. albicans* than the other *Candida* species of medical interest (Butler et al., 2009), as may be observed in Figure 2. This intimate evolutionary relationship is also evident in phenotypic and biochemical characteristics of both species. Phylogenetically, this pattern of evolution can be explained due to predominant clonal reproduction. However, with recombination events frequent enough to generate a population with similar characteristics (Wu et al., 2014).

## Identification

### Conventional methods for *Candida tropicalis* identification

*C. tropicalis* has been quite reasonably well-identified with phenotypic methods until the present moment (Table 1). This is contradictory to some other *Candida* spp., where molecular identification is mandatory due to the existence of cryptic species.

**Table 1:** Conventional methods used for *Candida tropicalis* laboratorial identification.

	**Method**	**Principle**	**Advantages**	**Disadvantages**	**References**
Classical methodology	Auxanogram and zymogram	Assimilation and fermentation of several different carbon and nitrogen souces	Easy execution and low cost	Laborious and time-consuming, subjectivity of interpretation	Pincus et al., 2007; Sariguzel et al., 2015
	Microculture on cornmeal agar containing Tween 80	Yeasts incubation on culture medium with Tween 80 and low oxygen tension esporulation and filamentation			
	Urease test	Urea hydrolysis alkalinizes the medium, causing the pH indicator to change. The medium goes from yellow to pink, indicating positivity			
Chromogenic media	Chromagar *Candida*®, *Candida* ID2®, CandiSelect4®, *Candida* Brilliance®	Different substrates react with specific enzymes of the main *Candida* species and induce the formation of colonies with different colors for tpresumptive identification	Rapid screening of different species and checking the purity of *Candida* colonies, detects mixed infections; high sensitivity and specificity	Presumptive identification for only five species of the *Candida* genus	Alfonso et al., 2010; Sariguzel et al., 2015; Zhao et al., 2016
Semi-automated methods	API 20C AUX, API ID 32C system	Galleries with different carbon sources, where growth and assimilation is observed by turbidity in the respective well	Good reproducibility and easy execution	May not be completely accurate on some cases and may lead to an incomplete identification, needing supplementary tests or even give a wrong identification for some species; higher cost. Not all the rare *Candida* species are included in the galleries	Bowman and Ahearn, 1976; Stefaniuk et al., 2016
	CandiFast® system	The identification well contains cycloheximide, besides seven carbohydrates, where fermentation is analyzed after acidification and alteration of media colors due to the presence of a pH indicator	Used for identification and antifungal susceptibility testing		Gündeş et al., 2001
	AuxaColor™ Kit	Assimilation of 13 sugars, besides the enzymatic detection of N-acetyl-galactosaminidase, phenoloxidase and L-proline arilamidase	Good reproducibility and easy execution		Pincus et al., 2007
Automated methods	Vitek2® System	Fluorometric and colorimetric methods for microorganism's identification and analysis in a software which contains a database with 52 yeast species	Rapid results, requires minimal preparation of reagents		Posteraro et al., 2015
	BD Phoenix™	Polystyrene strips contain three fluorescent control wells (a negative and two positives) with 47 wells containing lyophilized substrates			Grant et al., 2016

Although the classical methodology is of easy execution, it is very laborious and time-consuming making it difficult to be used in microbiology routine laboratories (Table 1; Pincus et al., 2007; Sariguzel et al., 2015).

The use of chromogenic media, with different substrates that react with specific enzymes of the main *Candida* species induce the formation of colonies with different colors and has been used for the presumptive identification of *C. tropicalis*. They have all been used for the screening of distinct species, besides being used to check the purity of *Candida* colonies and may be helpful to detect mixed infections. Quite a few number of different chromogenic culture media are currently commercially available for yeasts identification, and they have been successfully used for the initial screening of *C. tropicalis* colonies (Table 1).

Several commercially available kits used for yeasts identification based on carbohydrates used by oxidative pathways have been in the market in order to facilitate the process used for yeasts identification (Table 1). *C. tropicalis* identification with commercial methods have been performed since 1975; since then, several papers have been published in the literature evaluating the efficiency of this method. In a recent study by Stefaniuk and colaborators), the API ID32C system (bioMérieux) was used for the identification of 124 *Candida* clinical isolates, where 21 *C. tropicalis* isolates (100% of cases) were accurately identified (Stefaniuk et al., 2016). In a study performed by Alfonso et al., with 240 isolates of different *Candida* species, the authors found the accurate identification of 34 isolates of *C. tropicalis* with the API ID 32C system (Alfonso et al., 2010). Gundes et al. compared the efficiency of different commercial methods used in the identification 116 yeasts of medical interest, demonstrating the accuracy of 87% (101 out of 116) for API 20C® against 82.7% (96 out of 116) with Candifast® system. However, *C. tropicalis* was accurately identified in 100% of cases with both methods (Gündeş et al., 2001). The AuxaColor™ Kit (Bio-Rad) identification has been shown to be accurate in 63.8–95.2% of cases (Pincus et al., 2007). Recently, in a meta-analysis performed by Posteraro et al. (2015), including a total of 26 studies that evaluated yeasts identification methods, they observed that *C. tropicalis* was accurately identified in 168 out of 184 cases tested with AuxaColor™ and 55 out of 66 cases by using API ID32C® (Posteraro et al., 2015).

Besides semi-automated methods currently available, there are other methods completely automated for yeasts identification (Table 1). Won and collaborators performed a study that compared the efficiency of several medically important yeast species identification with the automated systems Vitek2® and BD Phoenix™. This study included a total of 341 isolates, from 49 species and *C. tropicalis* (36 isolates) was accurately identified in 34 cases with BD Phoenix™ System and in 32 occasions with Vitek2® (Won et al., 2014).

The conventional methods of identification including the classical methods, semi-automated and automated systems may not be completely accurate on some cases and may lead to an incomplete identification, needing supplementary tests or even give a wrong identification for some species (Marcos and Pincus, 2013; Chao et al., 2014). Therefore, molecular biology advances are of extreme importance for microorganism's identification because of the fact they are more accurate, and may reduce costs involving identification during the whole process, resulting in a decreased time for the release of results (Chao et al., 2014; Posteraro et al., 2015).

### Molecular methods and proteomics for the identification of *Candida tropicalis*

Recently, the evaluation of the protein profile of each species has been used as the basis for yeasts identification and has been proven as more efficient than the conventional methods (Santos et al., 2011; Chao et al., 2014; Stefaniuk et al., 2016). The protein profile by mass spectrophotometry is a simple methodology of easy sample preparation and short time for analysis (Table 2; Keceli et al., 2016).

**Table 2:** Molecular methods and proteomics for the identification and genotyping of *Candida tropicalis*.

	**Method**	**Principle**	**Advantages**	**Disadvantages**	**References**
Proteomics	Protein profile by mass spectrophotometry	Uses an ionizing matrix and has been assembled to automated methods of microorganisms identification such as MALDI Biotyper and VITEK-MS and several other mass spectrometer	Simple methodology of easy sample preparation and short time for analysis, more efficient than the conventional methods, accurate identification	Higher cost of equipment, necessity for specialized training; possible lack of a robust database	Chao et al., 2014; Angeletti et al., 2015; Panda et al., 2015; Sariguzel et al., 2015; Keceli et al., 2016; Stefaniuk et al., 2016
Molecular identification	Molecular rDNA sequencing	Based on the ability of DNA polymerase to copy a DNA strand from the template in the presence of a primer. The inclusion of fluorescent markers with different colors allows the differentiation of the chains truncated by the respective fluorescence	Robust technique, automated, Higher accuracy, gold standard identification	Requirement for specialized equipment, expensive reagents, and highly trained personnel	Pincus et al., 2007
	PNA-FISH	Based on the use peptide nucleic acid probes directed to specific rRNA species of the main *Candida* species tagged with fluorescent dyes	High sensitivity and specificity	There may be some problem in discriminating closely related microorganisms	Stender, 2003; Hall et al., 2012; Stone et al., 2013; Calderaro et al., 2014; Gorton et al., 2014; Aydemir et al., 2016
Genotyping	Randomly Amplified Polymorphic DNA (RAPD)	Based on the amplification of DNA fragments by polymerase chain reaction (PCR) by using shortprimers containing random sequences	Fast, simple and low-cost method for detecting polymorphisms; Does not require radioactively labeled probes; use of arbitrary primers, no need of initial genetic or genomic information, and the requirement of only tiny quantities of target DNA	Dominant technique; low reproducibility and low discriminatory power; difficult standardization, possible problems of interpretation	Wu et al., 2014; Almeida et al., 2015
	Microsatellites analysis	Based on the amplification by PCR of small tandem sequence repeats from 2 to 6 highly polymorphic nucleotides, present on chromosomal telomeric regions	Easy execution, reproducible, appropriate for large-scale epidemiological studies, good discriminatory power;	Technical challenges during the construction of enriched libraries and species-specific primers	
	Multilocus Sequence Typing (MLST)	Based on the amplification of 6–10 housekeeping genes by PCR, with further PCR products purification and gene sequencing. Gene sequencing generates the sequence type (ST) for haploid organisms and diploid sequence types (DST) for the diploids microorganisms, which also may be compared to a database	Robust technique with high discriminatory power, excellent reproducibility, easy standardization; data that can be shared and compared between different laboratories easily through the Internet	Requirement for specialized equipment, expensive reagents, and highly trained personnel; phylogenetic relationships and resolution of clones can be masked by the use of slowly evolving housekeeping genes	Maiden et al., 1998; Tavanti et al., 2005; Odds and Jacobsen, 2008; Chen et al., 2009; Wu et al., 2012

The accurate identification of *C. tropicalis* by proteomics analysis has been demonstrated in several studies which compared identification methods (Chao et al., 2014; Angeletti et al., 2015; Panda et al., 2015; Sariguzel et al., 2015; Keceli et al., 2016; Stefaniuk et al., 2016). *C. tropicalis* was accurately identified in 22/22 (100%) (Sow et al., 2015), in 21/21 (100%) (Stefaniuk et al., 2016), in 18/18 (100%) (Angeletti et al., 2015), in 17/17 (100%) (Chao et al., 2014), in 13/13 (100%) (Keceli et al., 2016), and in 2/2 (100%) by VITEK-MS (Sariguzel et al., 2015). The system performance of the MALDI Biotyper system also showed satisfactory results for the identification of *C. tropicalis*, where the accurate identification was found for 21/21 (100%) (Stefaniuk et al., 2016), 17/17 (100%), and in 18/18 (100%) (Angeletti et al., 2015) of cases.

Several studies have also been performed to evaluate PNA-FISH performance for different *Candida* species isolated from different anatomic sites, where conclusive results for *C. tropicalis* ranged from 96 to 100% of cases (Table 2; Hall et al., 2012; Stone et al., 2013; Calderaro et al., 2014; Gorton et al., 2014).

Although the methods used for microorganism's identification by using PNA-FISH and protein profile analysis using mass spectrophotometry techniques are accurate and have high sensitivity and specificity, molecular sequencing has been considered the gold standard technique for microorganisms identification recently (Keceli et al., 2016). rDNA ITS region sequencing has been quite satisfactorily used for *C. tropicalis* identification elsewhere. The main target for yeasts DNA molecular sequencing is the ribosomal (rDNA) region (Pincus et al., 2007). This region contains conserved domains separated by variable regions (the small sub unities 18S and 5.8S, besides the large subunit 26S, while these sub unities are separated by the interespacer regions ITS1 and ITS2) which contain species-specific sequences used as the preferential target for universal primers used of identification (Table 2; Merseguel et al., 2015; Shi et al., 2015; Benedetti et al., 2016).

### *Candida tropicalis* genotyping

Genotyping methods have largely been used recently to investigate a genetic correlation of different strains of the same species or even among different species (Table 2). These methods may be applied to the investigation of infections caused by similar or identical strains, besides the observation of possible micro-evolution or strains substitution during colonization and infection (da Costa et al., 2012; Almeida et al., 2015).

Recently, Almeida and collaborators employed RAPD technique with three different random primers (OPA-18, OPE-18, and P4) to evaluate the genetic variability of 15 clinical isolates of *C. tropicalis* obtained from patients with candiduria (Almeida et al., 2015). The analyses of the dendrogram constructed with DNA bands with the best discriminatory power primer (OPA-18) showed four well-defined clusters (I, II, III, and IV), where cluster I and II showed above 90% similarity among them, while clusters III and IV had 70% similarity.

Da Costa et al. (2012) genotyped by RAPD 15 strains of *C. tropicalis* oral isolates with primers OPA-01, OPA-09, OPB-11, OPE-18, and SEQ-06 (da Costa et al., 2012). OPA-01 showed the best discriminatory power, presenting ten distinct patterns for *C. tropicalis* isolates, with 80% similarity (da Costa et al., 2012). Another study using primers OPE-03, RP4-2, OPE-18, and AP50-with 12 catheter tip and urine isolates, obtained 9 different clusters with similarities coefficients (SABs) ranging from 0.8 to 1.0, where different strains were considered unrelated (if SAB was bellow 0.8), moderately related (SAB 0.8–0.89), highly related (SAB 0.90–0.99), and identical (SAB 1.0) (Marol and Yücesoy, 2008).

Almeida et al. (2015) typed 15 isolates of *C. tropicalis* with microsatellites and obtained the presence of five different alleles with the marker *URA3* and eight different allelic combinations with the *CT14* locus, being this marker considered to have a better discriminatory power than the *URA3* locus (Almeida et al., 2015).

By evaluating 65 clinical isolates of *C. tropicalis* obtained from different anatomic sites, Wu et al. used different markers of sequence tandem repeats, as follows: Ctrm1, Ctrm7, Ctrm10, Ctrm12, Ctrm15N, Ctrm21, Ctrm24, and Ctrm28 and selected six loci for population genetic analyses (Ctrm1, Ctrm10, Ctrm12, Ctrm21, Ctrm24 and Ctrm28), obtaining a total of 7 (Ctrm24 e Ctrm28) to 27 (Ctrm1) distinct genotypes (Wu et al., 2014).

The methodology known as MLST (Multilocus Sequence Typing) was originally described by Maiden et al. (1998). Therefore, by using MLST, strains from different geographic regions and various anatomic sources may be analyzed and compared. Strains maintenance, substitution, and multiple colonization may be investigated (Maiden et al., 1998; Chen et al., 2009; Wu et al., 2012, 2014).

The first MLST studies on *C. tropicalis* were performed in 2005, by Tavanti et al. with DNA sequencing of 6 housekeeping genes (*ICL1, MDR1, SAPT2, SAPT4, XYR1*, and *ZWF1*α). In this study, 106 isolates of *C. tropicalis* (104 human clinical isolates and 2 from animal origin) were evaluated, where 87 DSTs where obtained, grouped within three different highly related clades (Tavanti et al., 2005). In the study performed by Wu et al., with 58 strains of *C. tropicalis* from different anatomic sites by MLST, 52 different DSTs grouped within 6 different clades where obtained (Wu et al., 2012). Therefore, MLST is considered a very robust molecular technique used for typing with high discriminatory power, being widely used to evaluate intra-specific variability for different microorganisms including *C. tropicalis* (Tavanti et al., 2005; Odds and Jacobsen, 2008; Chen et al., 2009; Wu et al., 2012).

## Virulence factors

The ability of yeasts to adhere, infect, and cause diseases altogether is defined as a potential of virulence or pathogenicity. According to Cauchie et al. (in press), it was previously believed that species of the *Candida* genus were passively involved in the process of establishment of infection. However, it is now established that these yeasts play an active role in the infectious process through the action of several virulence factors (Cauchie et al., in press).

### Adhesion to epithelial and endothelial cells

Adhesion of blastoconidia to host cells is considered the first step for both colonization and the establishment of *Candida* infections and involves interactions between fungal cells and host surfaces (Cannon and Chaffin, 2001). It is a complex and multiphase process, including different factors, such as the microorganism involved, the composition of adhesion surfaces and several environmental factors (Silva-Dias et al., 2012).

Galan-Ladero et al. (2013) performed a study with 29 *C. tropicalis* isolates with hydrophobicity potential (Galan-Ladero et al., 2013). The cell wall structure is composed by hydrophobic proteins embedded in a cellular matrix which may favor the initial interaction, because hydrophobic particles tend to attach to a high variety of plastic materials and host proteins such as laminin, fibrinogen, and fibronectin (Tronchin et al., 2008).

Genes which codify proteins related to adhesion processes are differentially expressed, accordingly to a variety of hosts and environmental conditions (Sohn et al., 2006; Verstrepen and Klis, 2006). Despite the fact that *ALS* genes (Table 3) are highly involved with adhesion in *C. albicans*, it has been reported that several *Candida* species also have the ability to adhere to human buccal and vaginal epithelial cells, besides to the gastrointestinal epithelia of mice and several different plastic materials, motivating studies on adhesion in Non-*C. albicans Candida* (NCAC) species (Klotz et al., 1983).

**Table 3:** Genes recognized as virulence factors in *Candida tropicalis*.

	**Gene**	**Gene product in *C. tropicalis***	**Biological function**	**References**
Adhesion to epithelial cells	*ALS*	Als1 Als2 Als3	Adhesin	Hoyer et al., 2001; Punithavathy and Menon, 2012
	*HWP1*	Hwp1p	Hyphal cell wall adhesin	Wan Harun et al., 2013
Morphogenesis	*UME6*	Ume6p	Positive transcription regulator responsible for hyphae morphology and extension; induces *HGC1* transcription	Lackey et al., 2013
	*NRG1*	Nrg1p	Negative transcription regulator; inhibiting filamentation	
	*HGC1*	Hcg1p	Forms a complex between cycline/Cdk and CDC28 kinase, to inhibit cell separation and activation of Cdc42 regulator (involved in vesicular transport in hyphae and actin polymerization)	Zheng et al., 2007; Gonzalez-Novo et al., 2008; Lackey et al., 2013
	*PHR1*	Phr1p	Remodeling of the cell wall, necessary for maintenance of hyphae shape and growth, adhesion to abiotic surfaces and invasion of the epithelium	Calderon et al., 2010
	*CDC12*	cdc12p septin	Formation of the cytoskeleton during cell growth in filamentation; Binding to cdc3p actin ligand	Chang et al., 1997; Li et al., 2012
	*WOR1*	Wor1p	Transcription factor that induces filamentation	Slutsky et al., 1987; Porman et al., 2013
Phenotipc switching	*EFG1*	Efg1	Activator or a repressor of hypha formation	Mancera et al., 2015
	*WOR1*	Wor1p	Master regulator of the white-opaque switching	Slutsky et al., 1987; Porman et al., 2013
Biofim formation	*ALS*	Als1 Als2 Als3	Adhesin	Hoyer et al., 2001; Punithavathy and Menon, 2012; Wan Harun et al., 2013
	*HWP1*	Hwp1p	Hyphal cell wall adhesin	
	*BCR1*	Bcr1p	Transcription factor for regulation of adhesin production	
	*RBT5*	Rbt5p	Filamentation of cells in the biofilm	Nobile and Mitchell, 2006; Fitzpatrick et al., 2010
	*UME6*	Ume6p	Negative dispersion regulator of biofilm cells	
	*WOR1*	Wor1p	Negative regulator of mature biofilm cell release	Uppuluri et al., 2010
	*NRG1*	Nrg1p	Positive regulator of cells dispersion in biofilm	
	*ERG11*	Erg11p	Mechanisms of resistance	Lupetti et al., 2002
	*MDR1*	Mdr1p	Active drug efflux pump	Marie and White, 2009; Morschhäuser, 2010
Proteinase activity	*SAPT1 SAPT2 SAPT3 SAPT4*	Sapt1p	Protein hydrolysis	Togni et al., 1996; Zaugg et al., 2001; Silva et al., 2011
Phospholipases activity	*PLB1 PLC1*	Plb1, Plc1	Hydrolysis of ester bonds in glycerol phospholipids	Bennett et al., 1998; Hoover et al., 1998
Hemolytic activity	*RBT5*	Rbt5	GPI-anchored cell-wall protein involved in hemoglobin utilization	Nobile and Mitchell, 2006; Fitzpatrick et al., 2010

Punithavathy and Menon (2012) evaluated the presence of *ALS* genes in 48 isolates of *C. tropicalis* obtained from HIV-negative and positive patients. The authors found that 12 isolates (25%) expressed the *ALS1* gene, 24 isolates (50%) expressed *ALS2*, and 23 of them (48%) showed *ALS3* expression (Punithavathy and Menon, 2012).

*HWP1* (“Hyphal wall protein”) gene codify another important adhesin present on the hyphal cell wall (Table 3). *In vitro* studies demonstrated the presence of high amounts of Hwp1p at hyphal cell walls, while low amounts are present in blastoconidia (Naglik et al., 2006) and pseudo-hyphae (Snide and Sundstrom, 2006). The *HWP1* gene is involved in adhesion to human buccal epithelial cells (HBEC), codifying the first protein needed for biofilm formation (Sundstrom et al., 2002; Nobile et al., 2008).

The expression of this adhesin was recently reported for *C. tropicalis* in a study performed in Malaysia (Wan Harun et al., 2013) which investigated the presence of *HWP1* in NCAC species by using mRNA expression. *HWP1* mRNA transcription was positively regulated in *C. tropicalis*, indicating the ability of this species to express this adhesin. This study suggests that *HWP1* in *C. tropicalis* shares an identical sequence with *C. albicans*. Therefore, this is contradictory with the description of the presence of *HWP1* only in *C. albicans* (Ten Cate et al., 2009).

In fact, most of the studies report *C. albicans* as more adherent than other NCAC species, but *C*. *tropicalis* is considered the second most adherent species of the *Candida* genus (Calderone and Gow, 2002; Lyon and de Resende, 2006; Biasoli et al., 2010). For instance, Costa et al. evaluated the ability of adherence of *Candida* isolates obtained from the oral cavity of HIV individuals, patients with candidemia and catheter tips and found *C. albicans* as the most adherent species (average of 227.5 cells/100 HBEC) while *C. tropicalis* showed in average 123.5 cells/100 HBEC (Costa et al., 2010). Conversely, another study investigating adhesion by oral isolates of *C. albicans* and *C. tropicalis* to laminin and fibronectin detected by ELISA, reported *C. tropicalis* adhesion significantly higher than what was found for *C. albicans* (da Costa et al., 2012).

More recently, Menezes et al. (2013) evaluated the ability *Candida* spp. clinical isolates to adhere to glass cover slips (Menezes et al., 2013). They found higher adherence of *C. tropicalis* than *C. albicans* and yeasts belonging to the *C. parapsilosis* complex. A recent study performed in Brazil, with isolates from the oral cavity of kidney transplant recipients also demonstrated high ability of adherence to HBEC by *C. tropicalis* (Chaves et al., 2013). In this study, while *C. albicans* isolates showed about 237 cells/150 HBECs in average, an isolate of *C. tropicalis* had 335 cells/150 HBECs, reinforcing the remarkable role of adhesion as an important virulence factor in *C. tropicalis*.

### Morphogenesis and phenotypic switching

Subsequently to the adhesion step to host cells, bud-to-hyphae transition (also called morphogenesis) is highly relevant to some pathogenic yeasts, including *Candida* spp. (Calderone and Gow, 2002). It is one of the most important steps for the establishment of candidiasis and is considered a necessary step for several virulence processes, including invasion of host epithelial layers, endothelial rupture, survival to phagocytic cells attack, biofilm formation, and thigmotropism (Lackey et al., 2013).

Studies concerning morphogenesis are very well-established for *C. albicans*, with very well-established environmental signals, transcription regulators, and target genes involved in filamention (Gustin et al., 1998; Kumamoto and Vinces, 2005; Wapinski et al., 2007; Lackey et al., 2013). However, there are considerably less studies concerning morphogenesis in other NCAC species. Several *Candida* species may develop pseudo-hyphae, but quite a few are able to form true hypahe, including *C. albicans, C. dubliniensis*, and *C. tropicalis*. The latter do not show the same degree of filamentation than *C. albicans*; however, because of the fact they are frequently associated with infectious processes, they certainly have mechanisms of adaptation that may favor filamentation in specific environmental conditions (Lackey et al., 2013).

Galan-Ladero et al. (2013) evaluated the filamentation among *C. tropicalis* isolates obtained from different anatomic sites of patients admitted in a Spanish tertiary hospital (Galan-Ladero et al., 2013). The authors described high levels of filamentation for 76.6% of the isolates at the specific environmental conditions. Wapinski et al. (2007) reported that at least 55 out of the 105 genes involved in *C. albicans* filamentation are conserved in *C. tropicalis* (Wapinski et al., 2007).

Lackey et al. (2013) induced *C. tropicalis* cells filamentation and analyzed gene expression at the conditions provided (Lackey et al., 2013). They found significant filamentation in serum and glucose medium at 37°C. Optical microscopy showed the presence of elongated yeast-like cells, pseudohyphae, and true hyphae that were shorter than the ones found in *C. albicans*. They also verified that the negatively regulated gene *NRG1* has an important role in inhibiting filamentation in other NCAC species, suggesting that this gene may be related with poorer filamentation found among these species (Table 3). The *UME6* gene is transcriptionally induced during filamentation in *C. tropicalis*, similarly to what happens in *C. albicans* (Table 3; Banerjee et al., 2013).

Porman et al. (2013) reported the elevated expression of the transcriptional regulator *WOR1* (Table 3) in *C. tropicalis* cells cultivated on Spider medium (Porman et al., 2013). The micromorphological analysis of isolates with wrinkled phenotype showed that the most filamentous strains had *WOR1* overexpression. Wor1p homologs were also found in *Saccharomyces cerevisiae* (Cain et al., 2012) and *Histoplasma capsulatum* (Nguyen and Sil, 2008), controlling morphological transition within these species. This finding may suggest the existence of a common ancestor gene found in the *C. tropicalis* genome (Porman et al., 2013).

In addition, Wor1 which is the master regulator of the white-opaque switching, a phenomenon which is related to the reversible transition of cells from a white phase to an opaque phase, where cells are larger and elongated, while colonies have wrinkled appearance (Slutsky et al., 1987). Besides morphology, these two different cell types exhibit dramatic differences regarding to the preferred anatomic sites they colonize and infect, in addition to specific responses to environmental and nutritional signals and mating behavior (Mancera et al., 2015). In *C. tropicalis, WOR1* overexpression direct cells to the opaque phase which is involved in biofilm formation and morphogenesis (Porman et al., 2013).

It was described in the *C. tropicalis* genome an ortholog of the transcription factor Efg1 (enhanced filamentous growth), commonly found in *C. albicans* (Table 3; Mancera et al., 2015). The deletion of both alleles of the *EFG1* gene revealed that Efg1p is essential for filamentation, biofilm formation, and white-opaque switching in *C. tropicalis*, similarly to *C. albicans*, indicating conservation in the function of this ortholog gene.

Zhang Y. et al. (2016) reported a gray phenotype in *C. tropicalis* recently, whose cells are small and elongated, show intermediate mating competence and virulence in rats' animal models (Zhang Y. et al., 2016).

### Biofilm formation

The ability of yeast cells to form biofilms is an important determinant of virulence in *Candida* spp. and has been considered the main form of microbial growth recently (Donlan and Costerton, 2002; Fanning and Mitchell, 2012). Biofilms are complex structures formed by a community of microorganisms adhered to solid surfaces of either biotic or abiotic nature. Therefore, *in vitro* biofilm formation may be organized by three important steps, as follows: adhesion and colonization of yeast cells on a surface; cellular growth and proliferation, forming a basal layer; and pseudohyphal and/or true hyphal formation (for the species that are able to form filaments), with the subsequent secretion of an exopolymeric extracellular matrix which embeds microorganisms with low growth rates and altered phenotypes (Hawser and Douglas, 1995; Baillie and Douglas, 1999; Chandra et al., 2001; Ramage et al., 2001; Douglas, 2003). The exopolymeric matrix (EPS) may be secreted by different populations of either unique or multiple microbial species (Adam et al., 2002). Some advantages of biofilm formation include: the protection of microorganisms against environmental damage, nutrients availability, metabolic cooperation, and the acquisition of genetic modification (Douglas, 2002).

The formation of the microbial community involves a cascade of molecular mechanisms and fine alterations in gene expression (Nobile and Mitchell, 2006; Araújo et al., 2017). Signaling molecules which naturally occur in fungal cells as a response to environmental stimuli are part of this process present in the *Candida* genus (Ramage et al., 2006). This regulation is called “quorum sensing” (QS) mechanism and is the main communication form among several microorganisms correlated to population density (Albuquerque and Casadevall, 2012).

Farnesol is kind of self-regulator, a sesquiterpene with the ability to inhibit biofilm formation and altering the expression of 274 genes in *C. albicans*, specifically involved in filamentation. Weber et al. (2010) investigated the role of farnesol in biofilm formation of *C. tropicalis*. They found that besides inhibiting cellular aggregates, cells of the *C. tropicalis* mature biofilm were also influenced by farnesol, which may be related to their dispersion to other body sites (Nickerson et al., 2006; Ramage et al., 2006).

The initial step for biofilm formation is dependent of cellular adhesion cells to substrates and further formation of a basal layer (Nobile and Mitchell, 2006). *C. tropicalis* adhesins are also involved in biofilm formation (Table 3; Punithavathy and Menon, 2012; Wan Harun et al., 2013), and are regulated by the *BCR1* gene (also considered a cell wall regulator). In addition, the *RBT5* gene was also found in the *C. tropicalis* genome (Fitzpatrick et al., 2010).

Other genes involved in *C. tropicalis* biofilm formation are *WOR1, UME6, NRG1, ERG11*, and *MDR1* (Table 3). Besides being involved with morphogenesis and phenotypic switching, *WOR1* is one of the main transcriptional factors involved in biofilm formation (Xie et al., 2012; Porman et al., 2013).

*UME6* and *NRG1* are key transcription regulators directly involved in morphogenesis in *C. tropicalis* (Finkel and Mitchell, 2011). The overexpession of *UME6* reduces the liberation of mature sessile cells, while the decreased expression of *NRG1* promotes cells dispersion (Uppuluri et al., 2010).

With respect to the expression of resistance genes to antifungal drugs, *ERG11* (ergosterol biosynthesis), and *MDR1* (multidrug resistance) genes (Table 3) are related with resistance to fluconazole. Bizerra et al. (2008) reported the increased expression of these genes in sessile cells of *C. tropicalis* isolated from vulvovaginal candidiasis (VVC) and uroculture resistant to both fluconazole and amphotericin B. Punithavathy and Menon (2012) also demonstrated higher resistance to fluconazole of sessile cells liberated from mature biofilms of *C. tropicalis*.

There are evidences that biofilm cells formed on medical devices constantly released in the bloodstream guarantee the successful establishment of disseminated candidiasis (Fanning and Mitchell, 2012). Marcos-Zambrano et al. (2014) investigated biofilm formation in different *Candida* species obtained from episodes of fungemia and found *C. tropicalis* isolates were the strongest biofilm producers. In fact, another study reported that the high thickness of the EPS matrix of *C. tropicalis* biofilm cells may impair oxygen and nutrients diffusion to cells, and may be responsible for the lower metabolic activity (Alnuaimi et al., 2013).

Pannanusorn et al. (2013) also described *C. tropicalis* as the most efficient biofilm producers among bloodstream isolates as compared to other NCAC species. Paiva et al. (2012) evaluated the *in vitro* biofilm formation by *C. tropicalis* isolates obtained from VVC. This species was also considered the strongest biofilm producer compared to *C. albicans*, yeasts belonging to the *C. parapsilosis* complex, *C. glabrata*, and *C. guilliermondii*. A similar trend was also described by Udayalaxmi et al. (2014) with strains isolated from the urogenital tract (samples from vaginal fluid and urine) of patients from a tertiary hospital in the South of India. Therefore, *C. tropicalis* has been considered an important biofilm producer species of the *Candida* genus.

### Lytic enzymes

In order to facilitate host tissues invasions, several pathogenic microbes secrete lytic enzymes such as proteinases, phospholipases, and hemolysins to destroy, alter, or damage the integrity of host membranes, leading to the dysfunction, or rupture of host cells (Sanita et al., 2014).

Pathogenic *Candida* species produce a great variety of hydrolases, including secreted aspartic proteinases (Saps). These proteins have been intensely investigated, and possess a wide range of substrates, including collagen, queratin, and mucin. They have the ability to degrade epithelial barriers, antibodies, complement, and cytokines (Hube and Naglik, 2001), and are encoded by a great gene family. The *SAP* gene family is composed by 10 genes and was initially described in *C. albicans* (Ruchel et al., 1983). These genes are differentially regulated and expressed under several laboratory conditions and are activated during different stages of infections *in vivo*. In addition, some of the *SAP* genes are more important to superficial rather than systemic infections, and are also involved in other pathogenic process in *C. albicans*, such as adhesion, host tissue invasion, and immunological system cells evasion (Hube and Naglik, 2001).

It is well-known since 1983 that *C. tropicalis* is able to secrete proteinases as one of the most important determinants of virulence of this species (Macdonald and Odds, 1983; Ruchel et al., 1983). In 1991, Togni et al. reported the nucleotide sequence of a gene involved with the extracellular secretion of proteinases by this yeast, while in 1996 the same authors reported the secretion of Sapt1p by *C. tropicalis* (Togni et al., 1991, 1996). Subsequently, the crystallographic structure of this protein was published, and was considered very similar to the Sap2p of *C. albicans* (Symersky et al., 1997).

A study performed by Zaugg et al. (2001) suggested the existence of a *SAPT* gene family in the *C. tropicalis* genome, leading to four genes cloning: *SAPT* (1–4; Table 3). However, only Sapt1p was purified from culture supernatant and biochemically characterized.

Silva et al. (2011) investigated epithelial invasion by *C. tropicalis* using a reconstituted human buccal epithelia model. All the isolates tested were able to colonize this tissue and cause a great damage after 24 h. Real time PCR showed that *SAPT2-4* transcripts were detected, while *SAPT1* expression was rarely observed. In addition, the authors showed that there was no increase in *SAPT1* expression, suggesting that the high invasive capacity of *C. tropicalis* may not be related with the specific expression of this gene. Following the same trend, Togni et al. (1996) reported that *SAPT1* gene disruption in *C. tropicalis* seemed to have low effect in attenuation of virulence in mice, in a model of systemic infection.

Costa et al. (2010) evaluated proteinase activity of 15 isolates of *C. albicans* and 15 of *C. tropicalis* obtained from the saliva of dental patients in Brazil. All *C. tropicalis* isolates showed higher enzymatic production than *C. albicans*. These results are contradictory to most of the studies which suggest higher proteinase activity in *C. albicans* than in *C. tropicalis* (Zaugg et al., 2001; Sachin et al., 2012).

In addition to the secretion of proteinases, the secretion of phospholipases constitutes important determinants of virulence in *Candida* spp. This heterogeneous group of enzymes catalyzes the hydrolysis of ester bonds in glycerol phospholipids, with each enzyme participating in a specific reaction (Ghannoum, 2000). Secretion of phospholipases is therefore considered a key attribute for invasion of host epithelia, since phospholipids are major components of all cell membranes. In addition, the breakdown of these molecules promotes great instability in host cells, resulting in cellular lysis (Schaller et al., 2005).

One of the first studies that analyzed the production of phospholipases in *Candida* spp. was published in 1984 by Samaranayake et al. which demonstrated the secretion of these enzymes only in *C. albicans* isolates, without any detection in *C. tropicalis* (Samaranayake et al., 1984). However, other authors later reported phospholipase activity in isolates of this species. A recent study conducted by Jiang et al. (2016) with 52 strains of *C. tropicalis* found phospholipase activity in 31 isolates from different clinical sources. However, strains showed low enzyme production. Another study with 29 strains of several anatomic sites obtained from hospitalized patients, described low or no phospholipase activity in *C. tropicalis* (Galan-Ladero et al., 2010).

Conversely, a study conducted by Deorukhkar et al. (2014) investigating the expression of several virulence factors in 125 clinical isolates of this species concluded that the secretion of phospholipases was the main determinant of virulence expressed by these strains. The authors suggest that the variability of results between different authors may be a result of biological differences among the isolates tested.

Related to the expression of these enzymes in the presence of antifungal drugs, Anil and Samaranayake (2003) analyzed the effect of previous exposure of *C. albicans* and *C. tropicalis* to antifungal drugs on extracellular phospholipase activity. They concluded that the enzymatic activity of both species reduced significantly after previous exposure to nystatin and amphotericin B. In fact, they showed that *C. albicans* had greater phospholipase expression than *C. tropicalis*.

Phospholipases are classified into four major groups, named from A to D, all already well-described for *C. albicans* (Schaller et al., 2005). However, a few studies address this gene regulation in *C. tropicalis*. Phospholipase B (PLB; Table 3) is known to catalyze the hydrolytic cleavage of sn-1 and acyclic glycerophospholipid sn-2 esters (Ghannoum, 2000) and is primarily responsible for phospholipase activity in *C. albicans* (Schaller et al., 2005).

In 1998, Hoover et al. published an investigation with degenerate oligonucleotides (derived from conserved regions of the *PLB1* gene of *S. cerevisiae* and other fungi) to amplify homologous fragments of *PLB1* in *C. albicans* and *C. tropicalis* by PCR. The main PCR product obtained was a 540 bp fragment with a high probability of *PLB1*-correspondence of other fungi, and significant homology was found between the deduced amino acid sequence of the PCR product of *C. albicans* and *C. tropicalis* and the corresponding regions of *PLB1* sequence of *S. cerevisiae, Torulaspora delbrueckii*, and *Penicillium notatum* (~70–75% resemblance, ~55–65% identity). In that same year, Bennett et al. (1998) evaluated the presence of homologous sequences to C. *albicans PLC* in NCAC species, including five isolates of *C. tropicalis*. A DNA sequence homologous to *CAPLC1* was detected in only three of these isolates. Thus, the need for further studies addressing the molecular mechanisms related to phospholipase activity in *C. tropicalis* is evident.

The hemolysins are another group of proteins that significantly contribute for the dissemination of *Candida* infections, specifically in facilitating hyphal penetration in host tissues (Luo et al., 2004; Tsang et al., 2007). Hemolytic factors secreted by fungi cause hemoglobin liberation from red blood cells for further utilization by yeasts as an iron source (Giolo and Svidzinski, 2010). This chemical element is an essential cofactor to a great number of metabolic processes, such as oxygen transport, gene expression regulation, and DNA synthesis. Therefore, the ability of iron acquisition is of fundamental importance for microorganisms survival and establishment of infectious processes (Giolo and Svidzinski, 2010).

Manns et al. (1994) reported iron acquisition from erythrocytes by *C. albicans* as a consequence of a protein factor that promoted host cells lysis. In 1997, Tanaka et al. reported that this factor is liberated from the culture medium supernatant, and concluded that it was a cell wall manoprotein. The same phenomenon was observed in *C. tropicalis* (Favero et al., 2014), and although this factor is known as directly involved with yeasts pathogenicity, it is still poorly understood (Favero et al., 2011).

The study conducted by Luo et al. (2001) was the first one to show differences in hemolysin production by different *Candida* species on SDA plates containing sheep blood. The authors also observed that the hemolysis induced by this method could be divided into categories according to the standard microbiological nomenclature, including: total hemolysis (beta), partial hemolysis (alpha), or hemolysis absence (gama). In this study with 80 isolates of 14 different *Candida* species, all the five isolates of *C. tropicalis* showed a large clear halo around colonies, proving the ability of *C. tropicalis* in producing beta hemolysis. Similarly, Favero et al. (2011) detected hemolysin production in *C. tropicalis* strains after incubation in both solid and liquid SDA containing either human or sheep blood.

A study produced by Rossoni et al. (2013) evaluated the hemolytic activity in different *Candida* species obtained from the oral cavity of HIV positive patients. Strong hemolytic activity was observed in 75% of *C. tropicalis* isolates evaluated, only after *C. albicans*. Similar results were found for *Candida* isolates obtained from different anatomical sites (blood, synovial, and peritoneal liquid) where, again, *C. albicans* proceeded *C. tropicalis* in hemolysins production (de Melo Riceto et al., 2015).

Contradictory to these results, Favero et al. (2014) analyzing clinical *Candida* spp. isolates from bloodstream infection, reported low hemolytic activity in *C. albicans*, while *C. tropicalis* was the species tested with greater hemolysins production.

The genetic regulation of hemolysins production in the *Candida* genus was not still largely investigated (Anil et al., 2014). It is known that in *C. glabrata*, the *HLP* gene (hemolysin-like protein) encodes a protein associated with hemolytic activity (Luo et al., 2004). In *C. albicans* the Csap is involved with iron acquisition from host erythrocytes during hyphal development (Okamoto-Shibayama et al., 2014). This enzyme is a member of the Rbt5 protein (Table 3), also described in *C. tropicalis*, as previously mentioned. However, there are currently no studies in the literature concerning the genetic elucidation of hemolytic activity in *C. tropicalis*.

## *In vivo* models of infection by *Candida tropicalis*

The characterization of the expression of most variable virulence factors by *Candida* spp. and other fungi are necessary for the understanding each particular pathway involved in microorganisms pathogenicity (Takakura et al., 2003; Solis and Filler, 2012; de Campos Rasteiro et al., 2014). However, experiments performed *in vivo* involve different variables which cannot be controlled like what happens in experimental conditions *in vitro*, including the presence of body fluids, pH variation, commensal microorganisms, and their metabolites and host response during infection. Therefore, *in vivo* experimental models are needed for the global understanding of infectious disease pathogenicity, interactions with host cells and immune response as well as it is a more appropriate approach to evaluate new therapeutic strategies (Takakura et al., 2003; Solis and Filler, 2012; de Campos Rasteiro et al., 2014).

Several studies have been described in the literature with animal models of infections by *C. tropicalis* using mice (Bayegan et al., 2010; Mariné et al., 2010; Koga-Ito et al., 2011; Chen et al., 2014; Nash et al., 2016a; Wang et al., 2016; Zhang Y. et al., 2016). Nash et al. (2016a) evaluated the co-infection of six different *Candida* species (*C*. *albicans, C. tropicalis, C. parapsilosis, C. krusei, C. dubliniensis*, and *C. glabrata*) with *Staphylococcus aureus*, intraperitoneally inoculated. They evaluated mortality rates and attributed a score of 1–4 to evaluate characteristics of morbidity (creepy hair, absence of mobility, arched posture, and ocular secretion). *C. tropicalis* associated with *S. aureus* showed the second highest mortality rate (behind *C*. *albicans*) and a mortality index of 3 (Nash et al., 2016a).

Animal models of systemic infections may be induced by the inoculation of *C. tropicalis* via the lateral tail vein (Table 4; Zhang Y. et al., 2016). This via of infection was established by Zhang Y. et al. (2016) to evaluate virulence of a new phenotype described by *C. tropicalis*, the “Grey phenotype,” besides the other phenotypes already described (White–Opaque). After systemic infection through the tail vein with strains of each phenotype (Gray, White, and Opaque), animal organs have been removed and macerated and fungal load was evaluated. The authors found that cells of the Gray phenotype showed intermediate distribution, but greater than cells with the White phenotype for all the organs evaluated (kidney, lungs, spleen, liver, and brain), 24 h and 7 days after infection.

**Table 4:** *In vivo* models of *Candida tropicalis* infection.

**Organism**	**Site of infection**	**References**
Mice	Lateral tail vein	Zhang Q. et al., 2016
*Bombyx mori* larvae	Larval hemolymph	Hamamoto et al., 2004; Nwibo et al., 2015; Uchida et al., 2016
*Drosophila melanogaster* larvae	Injected in the thorax	Zanette and Kontoyiannis, 2013
*Galleria mellonella* larvae	Last left proleg	Forastiero et al., 2013

Other mice models of *Candida* infections have been described in the literature such as the VVC model described by Fidel et al. (1997), where doses of estradiol valerate are subcutaneously administered (0.1 mg/100 μl of sesame oil) in the vagina of animals infected with 5 × 10^4^ cells/20 μl PBS in order to successfully establish the vaginal infection (Fidel et al., 1996, 1997; Garvey et al., 2015; Nash et al., 2016b). Nevertheless, to the best of our knowledge, they were still not employed for the experimental investigation of VVC caused by *C. tropicalis*.

Alternative models of experimental infections have been broadly used for virulence and interactions with the host studies (Table 4; Hamamoto et al., 2004; Forastiero et al., 2013; Mesa-Arango et al., 2013; Zanette and Kontoyiannis, 2013; de Souza et al., 2015; Ishii et al., 2015; Shu et al., 2016). Several factors are considered as an advantage for the utilization of a model of infection using larvae, including an easier manipulation and lower maintenance cost (de Souza et al., 2015; Ishii et al., 2015). The Silkworm—*Bombyx mori*, (Lepidoptera: Bombycidae) produces a large enough larvae for antifungal drugs distribution studies (Nwibo et al., 2015; Uchida et al., 2016). *B. mori* larvae were used as a *C. tropicalis* model of infection in order to evaluate the effective dose of both fluconazole and amphotericin B (Hamamoto et al., 2004). When *C. tropicalis* was inoculated into the larval hemolymph followed by antifungal drugs administration, it was obtained an effective dose for 50% of them (ED 50%) of 1.8 μg/g of larvae for amphotericin B and fluconazole, being in agreement with previous animal models using mice previously performed (Hamamoto et al., 2004). Therefore, it confirms its possible use for *C. tropicalis* virulence studies.

*Drosophila melanogaster* larvae (Diptera: Drosophilidae), known as fruit flies also have been used as an animal model to study microbial interactions with innate immune response (Alarco et al., 2004). Zanette and Kontoyiannis satisfactorily used this model to investigate *C. tropicalis* strains with or without paradoxical growth (Zanette and Kontoyiannis, 2013).

*Galleria mellonella* larvae (Lepidoptera: Pyralidae) have been used as another invertebrate model to investigate fungal and host interactions (Champion et al., 2016), in systemic studies of antimicrobial efficiency (Wei et al., 2016), evaluation of virulence in immunosuppressive models (Torres et al., 2016), immunomodulatory response (Fuchs et al., 2016), and antifungal resistance (Souza et al., 2015).

*G. mellonella* infection by *C. tropicalis* was used to investigate cross resistance to azoles or multidrug resistance among them and amphotericin B (Forastiero et al., 2013). Two hours after infection with *C. tropicalis*, different antifungal drugs were applied (fluconazole, voriconazole, amphotericin B, and anidulafungin). In this study, 80% of the untreated infected larvae died between day 3 and 4 of infection, while better survival rates were observed for animals inoculated with susceptible strains (10 mg/kg/day of voriconazole and 9 mg/kg/day of fluconazol). When the larvae were infected with strains resistant to the azoles with the same therapeutic doses, survival rates were equivalent to the group that was untreated. This study demonstrates the reliable application of the use of the *G. mellonella* model for the study of infection by *Candida* as well as for the evaluation of antifungal action (Forastiero et al., 2013).

## Superficial and systemic infections

*C. tropicalis* belongs to the normal human microbiota and is present on the skin, gastrointestinal, genitourinary, and respiratory tracts of humans (Basu et al., 2003; Oksuz et al., 2007; Negri et al., 2010). This yeast has been associated with superficial and systemic infections all over the world, specifically in neutropenic patients, or in individuals with a reduction of the microbiota by antimicrobial use or presenting damage in gastrintestinal mucosa (Colombo et al., 2006).

*C. tropicalis* is classified as the third or fourth NCAC species more commonly isolated in the clinical practice (Pfaller et al., 2010; Peman et al., 2012), but it is considered the most prevalent yeast in Asia (Chakrabarti et al., 2009; Kothavade et al., 2010; Adhikary and Joshi, 2011) and the second or the third more isolated species in Brazil and other Latin America countries (20.9 and 13.2%, respectively) (Pfaller et al., 2010). The expressive increase in isolation of this yeast in cases of both superficial and systemic infections in different casuistic all over the world emphasizes its emergent character.

The clinical aspects of *Candida* infections may vary according with the body site affected. Oral candidiasis, VVC, and onychomycosis are superficial mycoses caused by this genus, while systemic candidiasis involves blood and deep-seated organs such as the lungs and gastrintestinal tract (Jacobs and Nall, 1990).

Oral candidiasis is an opportunistic infection caused by *Candida* commonly found in the eldery (due to low immunity caused by age), HIV patients, malnourished individuals and those submitted to systemic steroid therapy, denture wearers, and people with xerostomia (Muadcheingka and Tantivitayakul, 2015). Clinical manifestations are divided into white and erythematous forms. The white form is characterized by whitish lesions and includes pseudomembranous candidiasis and hyperplastic candidiasis. The erythematous form presents with red lesions, including acute atrophic candidiasis, chronic atrophic candidiasis, median rhomboid glossitis, angular cheilitis, and linear gingival erythema. There are also three forms which are not classified into these two clinical categories, which are chronic mucocutaneous candidiasis, cheilocandidiasis, and chronic multifocal candidiasis (Millsop and Fazel, 2016).

In Brazil, a study performed by da Silva-Rocha et al. (2014) investigated *Candida* species distribution of isolates obtained from the oral cavity of kidney transplant recipients from two geographic regions of Brazil (Northeast and South). The authors found that *C. tropicalis* was the second most prevalent species, corresponding to 4.5% of the isolates.

A prevalence study of *Candida* species obtained from oral candidiasis was carried out in Thailand with 250 strains isolated from 207 patients and *C. tropicalis* was the third most isolated species (10.4%) (Muadcheingka and Tantivitayakul, 2015). Similarly, in a study conducted in the northwest of Ethiopia with 215 oral cavity isolates from HIV positive patients, this yeast was also the third most prevalent species, with a percentage of isolation equal to 14.1%. More interestingly, 8% of them were resistant to fluconazole and 4% to ketoconazole, itraconazole, and fluocytosine (Mulu et al., 2013). Another Indian study concluded that there was a significant increase in *Candida* infections in oral cancer patients who underwent chemotherapy or radiotherapy, where NCAC species predominated, mainly *C. tropicalis*, occurring in 42.8% of cases (Jain et al., 2016).

VVC is an infection of the vulva and vagina caused by different *Candida* spp. (de Medeiros et al., 2014; Sobel, 2016). *C. tropicalis* is generally described as the third most prevalent *Candida* species in VVC, preceded by *C. albicans* and *C. glabrata* in most of the studies (Dias et al., 2011; Kanagal et al., 2014; Ragunathan et al., 2014).

A study developed in India by Vijaya et al. (2014) with 300 women of reproductive age with clinical signs of VVC reported *C. tropicalis* as the second most prevalent *Candida* species, corresponding to 26.4% of the isolates. Of these, 42.9% were resistant to fluconazole and 14.3% to voriconazole.

An investigation in Iran with 67 *Candida* isolates obtained from vaginal secretion samples from patients with VVC found that *C. tropicalis* was present in 5.9% of cases, with 100% resistance to fluconazole, 50% resistance to clotrimazole, 25% to ketoconazole, and 75% against terbinafine. In addition, all isolates showed dose-dependent susceptibility (DDS) to itraconazole (Salehei et al., 2012).

Nevertheless, *C. tropicalis* is reported to a lesser degree in cases of onychomycosis in relation to other species such as *C. albicans* and *C. parapsilosis* species complex, promoting paronychial infection mainly in immunosuppressed patients and individuals in extreme age (elderly and children) (Aghamirian and Ghiasian, 2010; Cambuim et al., 2011).

A study developed in South Korea reports the prevalence of the *Candida* genus in 59% of cases of onychomycosis in pediatric patients. The authors obtained 39 isolates, where only 2.6% of them belonged to *C. tropicalis* (Kim et al., 2013). Another study developed in Mexico analyzing 166 samples of dystrophic nails reports *C. tropicalis* as one of the less prevalent *Candida* species in onychomycosis, corresponding to 4.2% of the isolates. However, 14.2% were resistant to fluconazole, itraconazole, and ketoconazole (Manzano-Gayosso et al., 2011), reinforcing its clinical importance.

However, contradictory results were found in a Brazilian study with 200 *Candida* isolates obtained from nail infections that reported a prevalence of 26% for *C. tropicalis*. These authors also observed high antifungal drugs resistance in these isolates, including 30.6% resistance to fluconazole, 25% to itraconazole, 9.6% to ketoconazole, and 96.2% resistance to terbinafine (Figueiredo et al., 2007).

According to McCarty and Pappas (2016), invasive infection by *Candida* species is commonly associated with medical care, where they may be the third or fourth cause of bloodstream infection (BSI). Risk factors for systemic candidiasis are well-known and include the presence of central venous catheter (CVC), the exposure to broad spectrum antibacterial agents, prolonged staying in the ICU with or without mechanic ventilation (more than 3 days), complex surgery, the presence of necrotizing pancreatitis, hemodialysis, and immunosuppressive conditions (McCarty and Pappas, 2016). Candidemia caused by *C. tropicalis* infection has a greater association with skin petechia than other *Candida* species (Manzano-Gayosso et al., 2011), and this species was described as the most common etiological agent of invasive infection associated with the hospital environment in India (Giri and Kindo, 2012).

According to Kontoyiannis et al. (2001), *C. tropicalis* produces more persistent systemic infections than *C. albicans*, leading to a longer stay in the hospital environment. Other studies have associated *C. tropicalis* infections with a higher mortality rate when compared to other NCAC species, even when compared to *C. albicans* (Krcmery et al., 1999; Kontoyiannis et al., 2001; Eggimann et al., 2003). This factor may be related to the known higher virulence of both species as well as to a higher antifungal resistance by *C. tropicalis*.

Recently, an important multicenter study was carried out in 29 Spanish hospitals, where *C. tropicalis* was isolated in 7.6% of a total of 781 cases of candidemia and 20% of them were resistant to azoles (Guinea et al., 2014). Another multicenter study in China with 389 isolates from patients with candidemia admitted to intensive care units found *C. tropicalis* as the third most isolated species (17.2%), while resistance to fluconazole was observed in 37.3% of isolates of this species, as well as 10% of them were resistant to voriconazole (Liu et al., 2014).

A research carried out in Malaysia with 82 bloodstream isolates and peritoneal fluid reports *C. tropicalis* as responsible for 18.3% of the isolates obtained. Resistance to ketoconazole was observed in 20.9% of the clinical strains, in addition to 13.4% resistance to itraconazole (Santhanam et al., 2013). A study performed by Chang et al. (2013) with isolates from 152 cases of candidemia in Taiwan reported a prevalence of 19.7% for *C. tropicalis*.

In Brazil, a study conducted by Oliveira (2011) investigated candidemia in a pediatric hospital in Sao Paulo from 2007 to 2010. *C. tropicalis* was the second most isolated *Candida* species (24%), only preceded by *C. albicans*. More recently, a multicenter surveillance study involving 16 public and private hospitals in the five Brazilian regions (North, Northeast, Center-West, Southeast and South) was conducted, which investigated 137 episodes of systemic infections. NCAC species were responsible for 65.7% of the total infections and *C. tropicalis* was the third most isolated *Candida* species (15.3%) (Doi et al., 2016).

It is known that candidemia is the most common form of invasive candidiasis, but there are other less frequent clinical manifestations with *C. tropicalis* as an etiological agent (McCarty and Pappas, 2016). For example, a case of acute disseminated candidiasis in a pediatric patient with aplastic anemia (Fong et al., 1988); the formation of fungal vegetation in a mitral valve prosthesis, causing endocarditis (Nagaraja et al., 2005); and the development of septic arthritis in a cancer patient on chemotherapy with diabetes secondary to corticosteroid therapy that had a negative outcome (Vicari et al., 2003).

Another unusual clinical form of candidiasis is endophthalmitis, which is considered an important indicator of systemic infection in hospitalized patients (Donahue et al., 1994). *C. tropicalis* seems to be an important etiological agent of this infirmity, being classified as the fourth species of the genus to promote ocular infection in adult and pediatric patients attended at two medical centers in the USA (Dozier et al., 2011).

Disseminated chronic candidiasis is another condition of low occurrence characterized by the presence of histopathological evidence of candidiasis in the spleen, liver, and kidneys, or radiological evidence of hepatosplenic or renal candidiasis (Al-Anazi and Al-Jasser, 2006). Xu et al. (2010) described the isolation of *C. tropicalis* in a patient with acute leukemia whose computerized tomography showed multiple hypodense lesions in the liver and spleen. This yeast was also isolated from the kidneys of a patient diagnosed with acute lymphocytic leukemia (Sun et al., 2006) and was associated with a higher mortality rate than other *Candida* species involved in this disease (Al-Anazi and Al-Jasser, 2006).

Finally, *C. tropicalis* is more rarely found as an etiological agent of respiratory tract infections (Garczewska et al., 2016). This was the second most common yeast species in patients with cystic fibrosis, preceded only by *C. albicans*. Similarly, another study reports *C. tropicalis* as the second most common *Candida* species in cases of pulmonary co-infection with *Mycobacterium tuberculosis* (Kali et al., 2013).

## Antifungal susceptibility

The high incidence of severe infections caused by *C. tropicalis* has attracted attention, especially considering the evident increase in the reports of resistance of this yeast to antifungal drugs, which is a serious therapeutic problem.

Resistance to azoles in this species has been extensively reported, especially to fluconazole. In this respect, Anil and Samaranayake (2003) argue that the increasing global use of this drug is one of the main causes for the dominant tendency of infections caused by NCAC species to the detriment of *C. albicans*. It is known that there are several factors involved in the development of *Candida* spp. antifungal resistance in clinical settings, including indiscriminate antifungal therapy use in nosocomial infections (Joseph-Horne and Hollomon, 1997). However, studies on the molecular mechanisms underlying this phenomenon are still necessary.

With regard to azoles, the action target of these compounds is the enzyme 14 α-lanosterol demetilase (Erg11p), a product of the *ERG11* gene (Table 5), which is part of the ergosterol biosynthesis pathway (Lupetti et al., 2002). Ergosterol is the predominant component of the cell membrane of fungi, and influences various cellular functions such as membrane fluidity and integrity, as well as the adequate activity of various enzymes anchored to it, such as proteins related to nutrient transport and chitin synthesis. Therefore, the azoles cause depletion of ergosterol and accumulation of 14 α-methyl steroids harmful to cells, inhibiting growth of fungal cells (Lupetti et al., 2002).

**Table 5:** Genes involved with antifungal resistance mechanisms in *Candida tropicalis*.

	**Gene**	**Gene product in *C. tropicalis***	**Biological function**	**References**
Azoles	*ERG11*	Erg3p	Ergosterol biosynthesis pathway	Eddouzi et al., 2013; Vincent et al., 2013
	*ERG3*	Erg11p	Ergosterol biosynthesis pathway	Kelly et al., 1993; Manastir et al., 2011; Pam et al., 2012
	*MDR1*	Mdr1p	Energy-dependent transportation	Marie and White, 2009; Morschhäuser, 2010
	*CDR1*	Cdr1p	Energy-dependent transportation	
Amphotericin B	*ERG3*	Erg3p	Ergosterol biosynthesis pathway	Lupetti et al., 2002; Forastiero et al., 2013
	*ERG6*	Erg6p	Ergosterol biosynthesis pathway	Vandeputte et al., 2007
	*ERG11*	Erg11p	Ergosterol biosynthesis pathway	Forastiero et al., 2013
Echinocandins	*FKS1 FKS2 FKS3*	Fks1p	Catalytic action	Park et al., 2005; Garcia-Effron et al., 2008; Chen et al., 2011; Beyda et al., 2012; Jensen et al., 2013
	*RHO1*	Rho1p	Regulation of β-1,3-D-glucan biosynthesis and other cellular processes	

Sanglard and Odds (2002) report different mechanisms that may lead to resistance to azoles. The first is the action of multidrug transporters or efflux pumps, which leads to a decrease in drug concentration within the fungal cell (Pfaller, 2012). The positive regulation of *MDR1* (“multidrug resistance gene”) and *CDR1* (“*Candida* drug resistance”) genes (Table 5), are related to the active efflux of azoles in several *Candida* species, including *C. tropicalis* (Marie and White, 2009; Morschhäuser, 2010). The induction of efflux caused by *CDR* genes tends to affect all azoles. In contrast, efflux pumps encoded by *MDR* genes in *Candida* are normally selective for fluconazole (Pfaller, 2012).

Another pathway leading to azole resistance is the occurrence of amino acid substitutions in Erg11p, which is the target of these drugs, generating changes in protein conformation Forastiero et al., 2013). Increased *ERG11* gene expression results in the production of a large amount of 14 α-lanosterol demethylase, favoring the continuous synthesis of ergosterol, and the maintenance of cell integrity, which allows the fungus to resist the action of the drugs (Manastir et al., 2011). This factor may occur as a function of a point mutation in *ERG11* (Kelly et al., 1993). Pam et al. (2012) detected this point mutation in a *C. tropicalis* isolate with DDS to fluconazole and demonstrated increased *ERG11* expression.

Eddouzi et al. (2013) studied the molecular mechanisms of drug resistance in a clinical isolate of *C. tropicalis* with multidrug resistance to fluconazole, voriconazole, and amphotericin B, obtained from a hospital in Tunisia. Analysis of sterol production by mass spectrometry and gas chromatography revealed accumulation of 14α-methylfecosterol, 4,14α-dimethylzimosterol, and 14α-methyl-3β, 6α-diol, indicating change in Erg3p (Table 5). Another study reported the occurrence of *ERG3* mutation in a *C. tropicalis* isolate, with substitution of a phenylalanine for serine in portion 258, a residue that is absolutely conserved in this protein (Vincent et al., 2013).

A study conducted in five Chinese hospitals investigated resistance to these drugs in 52 clinical isolates of *C. tropicalis* (Jiang et al., 2013). Resistance to fluconazole was observed in 34.6% of the isolates, while 40.4% were resistant to itraconazole and only 7.7% to voriconazole. The authors suggest that voriconazole has a more potent activity against the clinical isolates of *C. tropicalis* than the other drugs tested.

Despite the number of studies involving strains of *C. tropicalis* resistant to azoles, there are still relatively less studies regarding the resistance of this species to other drugs, such as amphotericin B. This compound is the third most commonly used antifungal in clinical practice (Seneviratne et al., 2016) and is part of the class of polyenes. Its fungicidal activity comes from the ability to selectively bind to the ergosterol of the fungal cell, inducing the formation of pores in the plasma membrane, resulting in intense osmotic imbalance and rapid collapse of the cell (Brajtburg et al., 1990). A recent study reported that the production of reactive oxygen species is also part of the fungicidal mechanism of action of amphotericin B (Forastiero et al., 2013).

Amphotericin B resistance seems to be a rare phenomenon in yeasts, but Woods and Bard in 1974 demonstrated the development of resistance to this drug in two isolates of *C. tropicalis* obtained from the urine of a patient with pyelonephritis (Woods et al., 1974). A subsequent study of these strains revealed the existence of a mutation in the ergosterol of the cell membrane, exactly at the binding site of amphotericin B (Drutz and Lehrer, 1978). Also in the 1970s, Merz and Sandford reported the isolation of eight strains of *C. tropicalis* resistant to amphotericin B, obtained from urine of transplanted patients, with the same mutation in ergosterol (Merz and Sandford, 1979).

Reports of isolation of *C. tropicalis* resistant to this drug have been progressively increasing over the years. In 1988, Powderly et al., reported that the development of resistance to amphotericin B is most observed in patients with some kind of immunosuppression and who frequently use this drug (Powderly et al., 1988). Resistance to this polyene is believed to result from changes in ergosterol, or changes in the plasma membrane itself (Seneviratne et al., 2016).

Lupetti et al. (2002) postulated that resistance to amphotericin B in *Candida* species generally occurs due to defects in ergosterol biosynthesis and most likely results from mutations in the *ERG3* gene. In addition to the *ERG3* gene, mutations in *ERG6* can generate resistance to polyenes, a phenomenom already described in *C. tropicalis* (Vandeputte et al., 2007). A study conducted by Forastiero et al. (2013) showed that concomitant mutations in the *ERG11* and *ERG3* genes lead to multidrug resistance between amphotericin B and azoles (fluconazole and itraconazole) in *C. tropicalis*.

In addition to amphotericin B, echinocandins have been increasingly used in the treatment of invasive infections, being the first new class of echinocandins that target the fungal cell wall, blocking β-1,3-D-glucan synthase (Perlin, 2007). It has been described that these drugs have an excellent range of action against the main *Candida* species, including *C. tropicalis* (Pfaller et al., 2008).

A surveillance study carried out in 2008 by Pfaller et al. (2008) with 5,346 *Candida* spp. isolates obtained from candidemia infection showed a 12% prevalence for *C. tropicalis*, and all of them were 100% susceptible for the three echinocandins tested (caspofungin, anidulafungin, and micafungin).

Despite the extensive use of these drugs for more than a decade, the incidence of resistance in the *Candida* genus remains very low (Beyda et al., 2012). More recent surveillance studies have indicated an incidence of 2.9–3.1% *Candida* spp. resistance to echinocandins (Arendrup et al., 2010; Castanheira et al., 2010). However, recently Garcia-Effron et al. (2010) reported the isolation of three strains of *C. tropicalis* resistant to caspofungin obtained from patients with hematological malignancies. A study by Eschenauer et al. (2014) with 185 isolates of *C. tropicalis* reports 1.4% resistance to caspofungin, anidulafungin, and micafungin.

Another study described the presence of the paradoxical effect (or paradoxical growth) in 15 isolates of *C. tropicalis* in the presence of high concentrations of echinocandins (Soczo et al., 2007). This phenomenon was first documented by Stevens et al. (2004) for caspofungin in *C. albicans*, and is defined as fungal growth in the presence of echinocandin concentrations above MIC in broth microdilution susceptibility tests performed according to the guidelines of the Clinical Laboratory Standards Institute (CLSI, previously NCCLS) (Melo et al., 2007).

The target enzyme of echinocandins, called glucan synthetase, possesses at least two subunits: Fks1p (encoded by the *FKS1, FKS2* and *FKS3* genes) and Rho1p (Table 5; Beyda et al., 2012). Fks1p has a catalytic action and Rho1p is a regulatory protein of several cellular processes, including the biosynthesis of β-1,3-D-glucan (Chen et al., 2011). In general, the reduction of *C. tropicalis* susceptibility to echinocandins occurs by response to adaptive stress or mutations in the *FKS* genes.

With regard to adaptive responses, it is known that the fungal cell wall is a dynamic structure that presents a compensatory mechanism to increase the production of one or more components that are eventually inhibited, such as that produced by the action of echinocandins. A study by Chen et al. (2014) investigated the role of calcineurin in *C. tropicalis*, which is one of the main signaling pathways for the compensatory increase of chitin synthesis in *C. albicans*. Calcineurin is a phosphatase that regulates numerous stress response processes in fungi, including stress promoted on the cell wall (Cowen, 2009). The study demonstrated that, in fact, calcineurin is responsible for this effect on *C. tropicalis* against micafungin, since it promotes the thickening of the chitin layer of the cell wall as a function of β-1,3-D-glucan depletion.

In the case of mutations in the *FKS1* gene, it is already well-established that substitutions in specific gene regions cause reduced susceptibility to echinocandins, being quite associated with therapeutic failure (Perlin, 2007). Mutations in the *FKS1* gene in *C. tropicalis* were already described (Park et al., 2005). Garcia-Effron et al. (2008) demonstrated that 7.5% (3/40) of clinical isolates of *C. tropicalis* showed resistance to caspofungin because of amino acid substitutions in Fks1p. Jensen et al. (2013) also performed an investigation alterations in *FKS1*, with isolates of *C. tropicalis* from patients with acute lymphoblastic leukemia and found that after 8–8.5 weeks of treatment with caspofungin, two isolates showed resistance to the three echinocandins. Multilocus sequencing of *FKS1* revealed progressive development of heterozygosis, and finally the presence of homozygous mutation, leading to substitutions of amino acids S80P and S80S.

The low level of resistance of *C. tropicalis* to echinocandins and lower side effects, since they target the wall of the fungal cell, make them vital in cases of resistance to fluconazole and amphotericin B, with a broad spectrum of action against *C. tropicalis* (Pfaller et al., 2008).

## Natural products with antifungal properties against *Candida tropicalis*

Several groups have been dedicated to the study of products of natural origin with antifungal action, in order to identify and isolate compounds with effective activity, safety in use and low toxicity against pathogenic fungi (Correia et al., 2016).

There are several parts of the plants used to search for biological activity, with emphasis on antifungal action, such as leaves (Morais-Braga et al., 2016), stem bark (Mendes de Toledo et al., 2015), roots, seeds, and essential oils (Asdadi et al., 2015), that may be isolated used or in synergism with synthetic antifungal drugs, such as fluconazole (Mendes de Toledo et al., 2015).

The use of essential oils of *Vitex agnus* was used in a study by Asdadi et al. (2015) in clinical strains of *Candida* isolated from hospital infection. The extraction product was tested against *Candida* isolates using the principle of disc diffusion and broth macrodilution, according to the standardization of CLSI (Salari et al., 2016). It was observed that for the isolates of *C. tropicalis*, 10 μl of essential oils produced halos of inhibition of growth of 58 mm, superior to the halos of control drugs such as amphotericin B (8 mm), and fluconazole (21 mm) (Salari et al., 2016).

*Salvia rhytidoa Benth.*, A plant belonging to the family Lamiaceae, typical in Iran was used to evaluate the antifungal activity in several *Candida* isolates by Salari et al. (2016). A total of 96 clinical isolates of *Candida*, including 11 *C. tropicalis* strains were tested using broth microdilution with the methanolic extract, according to CLSI protocols (Salari et al., 2016). It was observed that for *C. tropicalis* the MIC range had a variation of 100–6.26 μg/ml. Similarly, Siqueira et al. found biological activity using a red propolis alcoholic extract, with an MIC range of 64–32 μg/ml (Siqueira et al., 2015) for this species.

In relation to plants found mainly in Brazilian territory, Correia et al. (2016) carried out an important study with different plants found in the Brazilian Cerrado, a region with an important number of species used in popular medicine, mainly in studies of essential oils with anti-*Candida* activity (Nordin et al., 2013; Correia et al., 2016).

In a study conducted by Morais-Braga et al. (2016) the interaction of aqueous and hydroethanolic extracts of *Psidium brownianum* was observed in association with fluconazole. The IC 50 values for fluconazole were 68.10 μg/ml for *C. tropicalis* CTINCQS 40042 and 41.11 μg/ml for *C. tropicalis* CTLM 23, obtained by broth microdilution. When in combination with fluconazole, the aqueous and hydroethanolic extracts of *P. brownianum* showed a significant reduction in IC 50 values, ranging from 37.2–3.10 μg/ml for CTINCM 40042 and 13.66–6.94 μg/ml for CTLM 23.

All these studies involving the evaluation of vegetal products with biological activity, especially against *C tropicalis*, has reinforced the great importance and necessity of the emergence of alternative and less toxic sources of treatment, alone or in combinations with different antifungal drugs in commercially available.

## *C. tropicalis* osmotic stress response and biotechnological applications

Several virulence attributes are expressed by fungi in response to stress conditions induced by the environment (Brown et al., 2014), and some yeasts can tolerate high salt concentrations, developing physical and genetic mechanisms to neutralize the two mains deleterious effects of osmotic stress, which are toxicity and loss of water and cellular turgidity (Garcia et al., 1997; Beales, 2004).

A study conducted by Rodriguez et al. (1996) reported the gene isolation involved with osmotic adaptation in *C. tropicalis*, a true homolog of *HAL3*, called Ct*HAL3*. In fact, *C. tropicalis* is able to grow in culture medium with more than 10–15% sodium chloride and has been isolated from the hypersaline environment for the first time from Dead Sea samples (Butinar et al., 2005). Bastos et al. (2000) reported the isolation of this yeast from a sample of Amazonian forest enriched with high salt concentration.

Garcia et al. (1997) carried out one of the few studies investigating the mechanisms of osmotic adaptation of *C. tropicalis*, analyzing ion extrusion. The results showed that Na^+^/K^+^-ATPase transporters are activated immediately after exposure to hypersaline environment, promoting rapid efflux of ions and restoring intracellular osmotic equilibrium.

Exposure to sodium chloride (NaCl) leads to high osmotic stress in fungal cells, promoting rapid loss of water that leads to reduced size and loss of cellular turgidity (Kühn and Klipp, 2012).

With regard to *C. tropicalis*, Garcia et al. (1997) reported that the accumulation of glycerol necessary for the restoration of a normal cellular physiology occurred only after the stationary phase. In addition, they found that there is a preponderant role of efflux pumps in the osmotic adaptation of *C. tropicalis* to the detriment of the nonionic compensatory mechanisms of water loss and turgor. Besides, the accumulation of intracellular glycerol seems to be less efficient than the activation of the Ion efflux pumps (Garcia et al., 1997).

Therefore, *C. tropicalis* is considered an osmotolerant yeast, since it can grow well in environments with high osmotic pressure, but this condition is not essential to its survival (Tokuoka, 1993). Such property is often associated with its use in industrial and biotechnological practices.

In the food industry, osmolytic strains of *C. tropicalis* improve xylitol production (Kwon et al., 2006; Misra et al., 2012). Rao et al. (2006) used *C. tropicalis* strains in hypersaline solution to produce xylitol from corn fiber and sugarcane bagasse. Another example of industrial application of this species is the production of ethanol from algae (Ra et al., 2015).

*C. tropicalis* is still widely used in bioremediation processes. Al-Araji et al. (2007) reported the use of this yeast in the commercial recovery of petroleum spillage. In 2011, Farag and Soliman reported the high degradability of crude oil and hydrocarbons by *C. tropicalis* (Farag and Soliman, 2011). Benard and Tuah (2016) also evaluated this property under conditions simulating sea water. In addition, Yan et al. (2005) demonstrated the high potential for degradation of phenol by *C. tropicalis* in saline medium. Microorganisms with this capacity are called biosorbents, found to correct pollution processes without causing damage to ecosystems (Leitão et al., 2007).

Halotolerance also provides a longer permanence of *C. tropicalis* in the coastal environment, allowing greater opportunity for contamination of bathers. Prolonged persistence in the marine environment may also lead to adaptation to high concentrations of other ions and UV light. This whole process can be reflected in genetic alterations that results in selection pressure (Krauke and Sychrova, 2008).

Recently, our group was involved in the investigation of osmotolerance and its relation to virulence expression *in vitro* with *C. tropicalis* isolated from the coastal environment. We found that these strains can fully express virulence attributes and may show a high persistence capacity on the coastal environment, because they all tolerated high salt concentration. In addition, they showed high MICs to several antifungal drugs used in current clinical practice, demonstrating that environmental isolates may have pathogenic potential and suggesting that the persistence of yeasts in the sand environment may have leaded to the overexpression of efflux pumps, that may partially explain the reason why *C. tropicalis* isolates not previously exposed to antifungal drugs had high levels of resistance to azoles and amphotericin B (Zuza-Alves et al., 2016).

## Concluding remarks

In conclusion, this review highlights important aspects of *C. tropicalis* biology and clinical relevance. This species may be easily identified by classical taxonomy, commercial, proteomics, and molecular methods and no cryptic sibling species has been discovered. This asexual yeast closely related to *C. albicans* may be considered of high virulence, which can be verified in animal models of superficial and systemics infections, plus its ability to form true hyphae and complex biofilm in *vitro*, besides the ability to secret proteinases, phospholipases and hemolisins. *C. tropicalis* is classified as the third or fourth NCAC species more commonly isolated in the clinical practice, while may be the second more frequently isolated *Candida* species in Latin America and Asia. Several mechanisms of antifungal resistance have been elucidated, including *ERG* and *FKS* gene families' mutations and efflux pumps. Some natural products have also been investigated as new potential use for future development of antifungal compounds active against *C. tropicalis*. This species is considered osmotolerant and this characteristic has been recently demonstrated to influence the expression of virulence factors and primary antifungal resistance. This ability to survive to high salt concentrations is a property that explains *C. tropicalis* potential use for biotechnological processes, including ethanol production through the fermentation of sea algae. Therefore, for all the factors previously described, *C. tropicalis* may be indubitably considered one of the most important *Candida* species.

## Author contributions

DZ and WS prepared the manuscript. GC designed all topics and revised the manuscript. All authors approved the final manuscript.

### Conflict of interest statement

The authors declare that the research was conducted in the absence of any commercial or financial relationships that could be construed as a potential conflict of interest.

## Abbreviations

*C. albicans*: *Candida albicans*
*C. tropicalis*: *Candida tropicalis*
CLSI: Clinical and Laboratory Standards Institute
MALDI-TOF/MS: Matrix-assisted laser desorption time-of-flight mass
ELISA: Enzyme-linked immunosorbent assay.

## References

[B1] Adam B. Baillie G. S. Douglas L. J. (2002). Mixed species biofilms of *Candida albicans* and *Staphylococcus epidermidis*. J. Med. Microbiol. 51, 344–349. 10.1099/0022-1317-51-4-344 11926741

[B2] Adhikary R. Joshi S. (2011). Species distribution and anti-fungal susceptibility of Candidaemia at a multi super-specialty center in Southern India. Indian J. Med. Microbiol. 29, 309–311. 10.4103/0255-0857.83920 21860117

[B3] Aghamirian M. R. Ghiasian S. A. (2010). Onychomycosis in Iran: epidemiology, causative agents and clinical features. Nihon Ishinkin Gakkai Zasshi 51, 23–29. 10.3314/jjmm.51.23 20185868

[B4] Al-Anazi K. Al-Jasser A. (2006). Candidaemia in patients with haematological disorders and stem cell transplant. Libyan J. Med. 1, 140–155. 10.3402/ljm.v1i2.4673 21526012 PMC3081354

[B5] Al-Araji L. Rahman R. N. Z. R. A. Basri M. Salleh A. B. (2007). Microbial surfactant. Asia Pac. J. Mol. Biol. Biotechnol. 15, 99–105.

[B6] Alarco A.-M. Marcil A. Chen J. Suter B. Thomas D. Whiteway M. (2004). Immune-deficient *Drosophila melanogaster*: a model for the innate immune response to human fungal pathogens. J. Immunol. 172, 5622–5628. 10.4049/jimmunol.172.9.5622 15100306

[B7] Albuquerque P. Casadevall A. (2012). Quorum sensing in fungi–a review. Med. Mycol. 50, 337–345. 10.3109/13693786.2011.652201 22268493 PMC4294699

[B8] Alfonso C. Lopez M. Arechavala A. del Perrone M. C. Guelfand L. Bianchi M. (2010). Presumptive identification of *Candida* spp. and other clinically important yeasts: usefulness of Brilliance *Candida* Agar. Rev. Iberoam. Micol. 27, 90–93. 10.1016/j.riam.2010.01.008 20346288

[B9] Almeida A. A. Nakamura S. S. Fiorini A. Grisolia A. B. Svidzinski T. I. Oliveira K. M. (2015). Genotypic variability and antifungal susceptibility of *Candida tropicalis* isolated from patients with candiduria. Rev. Iberoam. Micol. 32, 153–158. 10.1016/j.riam.2014.06.003 25766792

[B10] Alnuaimi A. D. O'Brien-Simpson N. M. Reynolds E. C. McCullough M. J. (2013). Clinical isolates and laboratory reference *Candida* species and strains have varying abilities to form biofilms. FEMS Yeast Res. 13, 689–699. 10.1111/1567-1364.12068 23927631

[B11] Angeletti S. Lo Presti A. Cella E. Dicuonzo G. Crea F. Palazzotti B.. (2015). Matrix-assisted laser desorption/ionization time of flight mass spectrometry (MALDI-TOF MS) and Bayesian phylogenetic analysis to characterize *Candida* clinical isolates. J. Microbiol. Methods 119, 214–222. 10.1016/j.mimet.2015.11.003 26551247

[B12] Anil S. Samaranayake L. (2003). Brief exposure to antimycotics reduces the extracellular phospholipase activity of *Candida albicans* and *Candida tropicalis*. Chemotherapy 49, 243–247. 10.1159/000072448 14504435

[B13] Anil S. Hashem M. Vellappally S. Patil S. Bandara H. M. Samaranayake L. P. (2014). Sub-inhibitory concentrations of antifungals suppress hemolysin activity of oral *Candida albicans* and *Candida tropicalis* isolates from HIV-infected individuals. Mycopathologia 178, 207–215. 10.1007/s11046-014-9802-0 25142726

[B14] Araújo D. Henriques M. Silva S. (2017). Portrait of *Candida* species biofilm regulatory network genes. Trends Microbiol. 25, 62–75. 10.1016/j.tim.2016.09.004 27717660

[B15] Arbour M. Epp E. Hogues H. Sellam A. Lacroix C. Rauceo. (2009). Widespread occurrence of chromosomal aneuploidy following the routine production of *Candida albicans* mutants. FEMS Yeast Res. 9, 1070–1077. 10.1111/j.1567-1364.2009.00563.x 19732157 PMC2784216

[B16] Arendrup M. C. Garcia-Effron G. Lass-Florl C. Lopez A. G. Rodriguez-Tudela J. L. Cuenca-Estrella M.. (2010). Echinocandin susceptibility testing of *Candida* species: comparison of EUCAST EDef 7.1, CLSI M27-A3, Etest, disk diffusion, and agar dilution methods with RPMI and isosensitest media. Antimicrob. Agents Chemother. 54, 426–439. 10.1128/AAC.01256-09 19884370 PMC2798528

[B17] Asdadi A. Hamdouch A. Oukacha A. Moutaj R. Gharby S. Harhar H.. (2015). Study on chemical analysis, antioxidant and *in vitro* antifungal activities of essential oil from wild *Vitex agnus-castus* L. *s*eeds growing in area of Argan Tree of Morocco against clinical strains of *Candida* responsible for nosocomial infections. J. Mycol. Med. 25, e118–e27. 10.1016/j.mycmed.2015.10.005 26611404

[B18] Aydemir G. Koc A. N. Atalay M. A. (2016). Evaluation of peptide nucleic acid fluorescent *in situ* hybridization (PNA FISH) method in the identifi cation of *Candida* species isolated from blood cultures. Mikrobiyol. Bull. 50, 293–299. 10.5578/mb.22092 27175502

[B19] Baillie G. S. Douglas L. J. (1999). Role of dimorphism in the development of *Candida albicans* biofilms. J. Med. Microbiol. 48, 671–679. 10.1099/00222615-48-7-671 10403418

[B20] Banerjee M. Uppuluri P. Zhao X. R. Carlisle P. L. Vipulanandan G. Villar C. C.. (2013). Expression of UME6, a key regulator of *Candida albicans* hyphal development, enhances biofilm formation via Hgc1- and Sun41-dependent mechanisms. Eukaryot. Cell 12, 224–232. 10.1128/EC.00163-12 23223035 PMC3571304

[B21] Bastos A. E. R. Moon D. H. Rossi A. Trevors J. T. Tsai S. M. (2000). Salt-tolerant phenol-degrading microorganisms isolated from Amazonian soil samples. Arch. Microbiol. 174, 346–352. 10.1007/s002030000216 11131025

[B22] Basu S. Gugnani H. C. Joshi S. Gupta N. (2003). Distribution of *Candida* species in different clinical sources in Delhi, India, and proteinase and phospholipase activity of *Candida albicans* isolates. Rev. Iberoam. Micol. 20, 137–140. 15456350

[B23] Bayegan S. Majoros L. Kardos G. Kemény-Beke A. Miszti C. Kovacs R.. (2010). *In vivo* studies with a *Candida tropicalis* isolate exhibiting paradoxical growth *in vitro* in the presence of high concentration of caspofungin. J. Microbiol. 48, 170–173. 10.1007/s12275-010-9221-y 20437148

[B24] Beales N. (2004). Adaptation of microorganisms to cold temperatures, weak acid preservatives, low pH, and osmotic stress: a review. Compr. Rev. Food Sci. Food Saf. 3, 1–20. 10.1111/j.1541-4337.2004.tb00057.x 33430556

[B25] Benard L. D. Tuah P. M. (2016). Biodegradation of sabah light crude oil by locally isolated *Candida tropicalis* RETL-Cr1 and *Pseudomonas aeruginosa* BAS-Cr1. Trans. Sci. Technol. 3, 101–106.

[B26] Benedetti V. P. Savi D. C. Aluizio R. Adamoski D. Kava-Cordeiro V. Galli-Terasawa L. V. (2016). Analysis of the genetic diversity of *Candida* isolates obtained from diabetic patients and kidney transplant recipients. Mem. Inst. Oswaldo Cruz 111, 417–422. 10.1590/0074-02760160042 PMC4957493 27276363

[B27] Bennett D. E. McCreary C. E. Coleman D. C. (1998). Genetic characterization of a phospholipase C gene from *Candida albicans*: presence of homologous sequences in *Candida* species other than *Candida albicans*. Microbiology 144, 55–72. 10.1099/00221287-144-1-55 9467900

[B28] Berman J. Hadany L. (2012). Does stress induce (para) sex? Implications for *Candida albicans* evolution. Trends Genet. 28, 197–203. 10.1016/j.tig.2012.01.004 22364928 PMC3340477

[B29] Beyda N. D. Lewis R. E. Garey K. W. (2012). Echinocandin resistance in *Candida* species: mechanisms of reduced susceptibility and therapeutic approaches. Ann. Pharmacother. 46, 1086–1096. 10.1345/aph.1R020 22811350

[B30] Biasoli M. S. Tosello M. E. Luque A. G. Magaro H. M. (2010). Adherence, colonization and dissemination of *Candida* dubliniensis and other *Candida* species. Med. Mycol.. 48, 291–297. 10.1080/13693780903114942 19626546

[B31] Bizerra F. C. Nakamura C. V. de Poersch C. Estivalet Svidzinski T. I. Borsato Quesada R. M. Goldenberg S.. (2008). Characteristics of biofilm formation by *Candida tropicalis* and antifungal resistance. FEMS Yeast Res. 8, 442–450. 10.1111/j.1567-1364.2007.00347.x 18248413

[B32] Blandin G. Ozier-Kalogeropoulos O. Wincker P. Artiguenave F. Dujon B. (2000). Genomic exploration of the hemiascomycetous yeasts: 16. *Candida tropicalis*. FEBS Lett. 487, 91–94. 10.1016/S0014-5793(00)02287-0 11152891

[B33] Bouchonville K. Forche A. Tang K. E. Selmecki A. Berman J. (2009). Aneuploid chromosomes are highly unstable during DNA transformation of *Candida albicans*. Eukaryot. Cell 8, 1554–1566. 10.1128/EC.00209-09 19700634 PMC2756872

[B34] Bowman P. I. Ahearn D. G. (1976). Evaluation of commercial systems for the identification of clinical yeast isolates. J. Clin. Microbiol. 4, 49–53. 956362 10.1128/jcm.4.1.49-53.1976 PMC274388

[B35] Brajtburg J. Powderly W. G. Kobayashi G. S. Medoff G. (1990). Amphotericin B: current understanding of mechanisms of action. Antimicrob. Agents Chemother. 34, 183–188. 2183713 10.1128/aac.34.2.183 PMC171553

[B36] Brown A. J. Budge S. Kaloriti D. Tillmann A. Jacobsen M. D. Yin Z.. (2014). Stress adaptation in a pathogenic fungus. J. Exp. Biol. 217, 144–155. 10.1242/jeb.088930 24353214 PMC3867497

[B37] Butinar L. Santos S. Spencer-Martins I. Oren A. Gunde-Cimerman N. (2005). Yeast diversity in hypersaline habitats. FEMS Microbiol. Lett. 244, 229–234. 10.1016/j.femsle.2005.01.043 15766773

[B38] Butler G. Rasmussen M. D. Lin M. F. Santos M. A. Sakthikumar S. Munro C. A.. (2009). Evolution of pathogenicity and sexual reproduction in eight *Candida* genomes. Nature 459, 657–662. 10.1038/nature08064 19465905 PMC2834264

[B39] Cain C. W. Lohse M. B. Homann O. R. Sil A. Johnson A. D. (2012). A conserved transcriptional regulator governs fungal morphology in widely diverged species. Genetics 190, 511–521. 10.1534/genetics.111.134080 22095082 PMC3276625

[B40] Calderaro A. Martinelli M. Motta F. Larini S. Arcangeletti M. C. Medici M. C.. (2014). Comparison of peptide nucleic acid fluorescence *in situ* hybridization assays with culture-based matrix-assisted laser desorption/ionization-time of flight mass spectrometry for the identification of bacteria and yeasts from blood cultures and cerebrospinal fluid cultures. Clin. Microbiol. Infect. 20, 468–475. 10.1111/1469-0691.12490 24304149

[B41] Calderone R. A. Gow N. A. (2002). Host recognition by *Candida* species, in Candida and Candidiasis, ed Calderone R. A. (Washington, DC: ASM Press), 67–86.

[B42] Calderon J. Zavrel M. Ragni E. Fonzi W. A. Rupp S. Popolo L. (2010). *PHR1*, a pH-regulated gene of *Candida albicans* encoding a glucan-remodelling enzyme, is required for adhesion and invasion. Microbiology 156, 2484–2494. 10.1099/mic.0.038000-0 20430812

[B43] Cambuim I. I. Macedo D. P. Delgado M. de Lima K. M. Mendes G. P. Souza-Motta C. M.. (2011). Clinical and mycological evaluation of onychomycosis among Brazilian HIV/AIDS patients. Rev. Soc. Bras. Med. Trop. 44, 40–42. 10.1590/S0037-86822011000100010 21340406

[B44] Cannon R. D. Chaffin W. L. (2001). Colonization is a crucial factor in oral candidiasis. J. Dent. Educ. 65, 785–778. 11518251

[B45] Castanheira M. Woosley L. N. Diekema D. J. Messer S. A. Jones R. N. Pfaller M. A. (2010). Low prevalence of fks1 hot spot 1 mutations in a worldwide collection of *Candida* strains. Antimicrob. Agents Chemother. 54, 2655–2659. 10.1128/AAC.01711-09 20368396 PMC2876398

[B46] Castellani A. (1912). Observations on the fungi found in tropical bronchomycosis. Lancet 179, 13–15. 10.1016/S0140-6736(00)51698-5

[B47] Cauchie M. Desmet S. Lagrou K. (in press). *Candida* and its dual lifestyle as a commensal a pathogen. Res. Microbiol. 10.1016/j.resmic.2017.02.005 28263903

[B48] Chakrabarti A. Chatterjee S. S. Rao K. L. Zameer M. M. Shivaprakash M. R. Singhi S.. (2009). Recent experience with fungaemia: change in species distribution and azole resistance. Scand. J. Infect. Dis. 41, 275–284. 10.1080/00365540902777105 19229762

[B49] Champion O. L. Wagley S. Titball R. W. (2016). Galleria mellonella as a model host for microbiological and toxin research. Virulence 7, 840–845. 10.1080/21505594.2016.1203486 27362761 PMC5029293

[B50] Chandra J. Kuhn D. M. Mukherjee P. K. Hoyer L. L. McCormick T. Ghannoum M. A. (2001). Biofilm formation by the fungal pathogen *Candida albicans*: development, architecture, and drug resistance. J. Bacteriol. 183, 5385–5394. 10.1128/JB.183.18.5385-5394.2001 11514524 PMC95423

[B51] Chang F. Drubin D. Nurse P. (1997). cdc12p, a protein required for cytokinesis in fission yeast, is a component of the cell division ring and interacts with profilin. J. Cell Biol. 137, 169–182. 10.1083/jcb.137.1.169 9105045 PMC2139860

[B52] Chang T. P. Ho M. W. Yang Y. L. Lo P. C. Lin P. S. Wang A. H.. (2013). Distribution and drug susceptibilities of *Candida* species causing candidemia from a medical center in central Taiwan. J. Infect. Chemother. 19, 1065–1071. 10.1007/s10156-013-0623-8 23732308

[B53] Chao Q. T. Lee T. F. Teng S. H. Peng L. Y. Chen P. H. Teng L. J.. (2014). Comparison of the accuracy of two conventional phenotypic methods and two MALDI-TOF MS systems with that of DNA sequencing analysis for correctly identifying clinically encountered yeasts. PLoS ONE 9:e109376. 10.1371/journal.pone.0109376 25330370 PMC4199611

[B54] Chaves G. M. Diniz M. G. da Silva-Rocha W. P. de Souza L. B. Gondim L. A. Ferreira M. A.. (2013). Species distribution and virulence factors of *Candida* spp. isolated from the oral cavity of kidney transplant recipients in Brazil. Mycopathologia 175, 255–263. 10.1007/s11046-013-9640-5 23539354

[B55] Chen K.-W. Chen Y.-C. Lin Y.-H. Chou H.-H. Li S.-Y. (2009). The molecular epidemiology of serial *Candida tropicalis* isolates from ICU patients as revealed by multilocus sequence typing and pulsed-field gel electrophoresis. Infect. Genet. Evol. 9, 912–920. 10.1016/j.meegid.2009.06.011 19540937

[B56] Chen S. C. Slavin M. A. Sorrell T. C. (2011). Echinocandin antifungal drugs in fungal infections: a comparison. Drugs 71, 11–41. 10.2165/11585270-000000000-00000 21175238

[B57] Chen Y. L. Yu S. J. Huang H. Y. Chang Y. L. Lehman V. N. Silao F. G.. (2014). Calcineurin controls hyphal growth, virulence, and drug tolerance of *Candida tropicalis*. Eukaryot. Cell 13, 844–854. 10.1128/EC.00302-13 24442892 PMC4135728

[B58] Choi M. J. Won E. J. Shin J. H. Kim S. H. Lee W. G. Kim M. N.. (2016). Resistance mechanisms and clinical features of fluconazole-nonsusceptible *Candida tropicalis* isolates compared with fluconazole-less-susceptible isolates. Antimicrob. Agents Chemother. 60, 3653–3661. 10.1128/AAC.02652-15 27044550 PMC4879413

[B59] Colombo A. L. Nucci M. Park B. J. Nouer S. A. Arthington-Skaggs B. da Matta D. A.. (2006). Epidemiology of candidemia in Brazil: a nationwide sentinel surveillance of candidemia in eleven medical centers. J. Clin. Microbiol. 44, 2816–28123. 10.1128/JCM.00773-06 16891497 PMC1594610

[B60] Correia A. F. Silveira D. Fonseca-Bazzo Y. M. Magalhães P. O. Fagg C. W. da Silva E. C.. (2016). Activity of crude extracts from Brazilian cerrado plants against clinically relevant *Candida* species. BMC Complement. Altern. Med. 16:203. 10.1186/s12906-016-1164-3 27401815 PMC4940766

[B61] Costa C. R. Passos X. S. de Souza L. K. de Lucena P. A. de Fernandes O. F. da Silva M. R. (2010). Differences in exoenzyme production and adherence ability of *Candida* spp. isolates from catheter, blood and oral cavity. Rev. Inst. Med. Trop. Sao Paulo 52, 139–143. 10.1590/S0036-46652010000300005 20602023

[B62] Cowen L. E. (2009). Hsp90 orchestrates stress response signaling governing fungal drug resistance. PLoS Pathog. 5:e1000471. 10.1371/journal.ppat.1000471 19714223 PMC2726949

[B63] da Costa K. R. Ferreira J. C. Lavrador M. A. Baruffi M. D. Candido R. C. (2012). Virulence attributes and genetic variability of oral *Candida albicans* and *Candida tropicalis* isolates. Mycoses 55, e97–e105. 10.1111/j.1439-0507.2011.02125.x 22035510

[B64] da Silva-Rocha W. P. Lemos V. L. Svidizisnki T. I. Milan E. P. Chaves G. M. (2014). *Candida* species distribution, genotyping and virulence factors of *Candida albicans* isolated from the oral cavity of kidney transplant recipients of two geographic regions of Brazil. BMC Oral Health 14:20. 10.1186/1472-6831-14-20 24628850 PMC3995545

[B65] de Campos Rasteiro V. M. da Costa A. C. B. P. Araújo C. F. De Barros P. P. Rossoni R. D. Anbinder A. L.. (2014). Essential oil of Melaleuca alternifolia for the treatment of oral candidiasis induced in an immunosuppressed mouse model. BMC Complement. Altern. Med. 14:1. 10.1186/1472-6882-14-489 25510285 PMC4301879

[B66] de Medeiros M. A. P. de Melo A. P. V. Gonçalves S. S. Milan E. P. Chaves G. M. (2014). Genetic relatedness among vaginal and anal isolates of *Candida albicans* from women with vulvovaginal candidiasis in north-east Brazil. J. Med. Microbiol. 63, 1436–1445. 10.1099/jmm.0.076604-0 25187602

[B67] de Melo Riceto É. B. de Paula Menezes R. Penatti M. P. A. dos Santos Pedroso R. (2015). Enzymatic and hemolytic activity in different *Candida* species. Rev. Iberoam. Micol. 32, 79–82. 10.1016/j.riam.2013.11.003 24704439

[B68] de Souza P. C. Morey A. T. Castanheira G. M. Bocate K. P. Panagio L. A. Ito F. A.. (2015). Tenebrio molitor (Coleoptera: Tenebrionidae) as an alternative host to study fungal infections. J. Microbiol. Methods 118, 182–186. 10.1016/j.mimet.2015.10.004 26453946

[B69] Deorukhkar S. C. Saini S. Mathew S. (2014). Virulence factors contributing to pathogenicity of *Candida tropicalis* and its antifungal susceptibility profile. Int. J. Microbiol. 2014:456878. 10.1155/2014/456878 24803934 PMC3996979

[B70] Dias L. B. de Souza Carvalho Melhem M. Szeszs M. W. Filho J. M. Hahn R. C. (2011). *Vulvovaginal candidiasis* in Mato Grosso, Brazil: pregnancy status, causative species and drugs tests. Braz. J. Microbiol. 42, 1300–1307. 10.1590/S1517-83822011000400009 24031756 PMC3768752

[B71] Diezmann S. Cox C. J. Schonian G. Vilgalys R. J. Mitchell T. G. (2004). Phylogeny and evolution of medical species of *Candida* and related taxa: a multigenic analysis. J. Clin. Microbiol. 42, 5624–5635. 10.1128/JCM.42.12.5624-5635.2004 15583292 PMC535224

[B72] Doi A. M. Pignatari A. C. Edmond M. B. Marra A. R. Camargo L. F. Siqueira R. A.. (2016). Epidemiology and microbiologic characterization of nosocomial candidemia from a Brazilian national surveillance program. PLoS ONE 11:e0146909. 10.1371/journal.pone.0146909 26808778 PMC4726651

[B73] Doi M. Homma M. Chindamporn A. Tanaka K. (1992). Estimation of chromosome number and size by pulsed-field gel electrophoresis (PFGE) in medically important *Candida* species. J. Gen. Microbiol. 138, 2243–2251. 10.1099/00221287-138-10-2243 1479351

[B74] Donahue S. P. Greven C. M. Zuravleff J. J. Eller A. W. Nguyen M. H. Peacock J. E.. (1994). Intraocular candidiasis in patients with candidemia: clinical implications derived from a prospective multicenter study. Ophthalmology 101, 1302–1309. 10.1016/S0161-6420(94)31175-4 8035995

[B75] Donlan R. M. Costerton J. W. (2002). Biofilms: survival mechanisms of clinically relevant microorganisms. Clin. Microbiol. Rev. 15, 167–193. 10.1128/CMR.15.2.167-193.2002 11932229 PMC118068

[B76] Douglas L. J. (2002). Medical importance of biofilms in *Candida* infections. Rev. Iberoam. Micol. 19, 139–143. 12825991

[B77] Douglas L. J. (2003). *Candida* biofilms and their role in infection. Trends Microbiol. 11, 30–36. 10.1016/S0966-842X(02)00002-1 12526852

[B78] Dozier C. C. Tarantola R. M. Jiramongkolchai K. Donahue S. P. (2011). Fungal eye disease at a tertiary care center: the utility of routine inpatient consultation. Ophthalmology 118, 1671–1676. 10.1016/j.ophtha.2011.01.038 21550121

[B79] Drutz D. J. Lehrer R. I. (1978). Development of amphotericin B-resistant *Candida tropicalis* in a patient with defective leukocyte function. Am. J. Med. Sci. 276, 77–92. 727219

[B80] Eddouzi J. Parker J. E. Vale-Silva L. A. Coste A. Ischer F. Kelly S.. (2013). Molecular mechanisms of drug resistance in clinical *Candida* species isolated from Tunisian hospitals. Antimicrob. Agents Chemother. 57, 3182–3193. 10.1128/AAC.00555-13 23629718 PMC3697321

[B81] Eggimann P. Garbino J. Pittet D. (2003). Epidemiology of *Candida* species infections in critically ill non-immunosuppressed patients. Lancet Infect. Dis. 3, 685–702. 10.1016/S1473-3099(03)00801-6 14592598

[B82] Eschenauer G. A. Nguyen M. H. Shoham S. Vazquez J. A. Morris A. J. Pasculle W. A.. (2014). Real-world experience with echinocandin MICs against *Candida* species in a multicenter study of hospitals that routinely perform susceptibility testing of bloodstream isolates. Antimicrob. Agents Chemother. 58, 1897–1906. 10.1128/AAC.02163-13 24395235 PMC4023707

[B83] Fanning S. Mitchell A. P. (2012). Fungal biofilms. PLoS Pathog. 8:e1002585. 10.1371/journal.ppat.1002585 22496639 PMC3320593

[B84] Farag S. Soliman N. A. (2011). Biodegradation of crude petroleum oil and environmental pollutants by *Candida tropicalis* strain. Braz. Arch. Biol. Technol. 54, 821–830. 10.1590/S1516-89132011000400023

[B85] Favero D. Franca E. J. Furlaneto-Maia L. Quesada R. M. Furlaneto M. C. (2011). Production of haemolytic factor by clinical isolates of *Candida tropicalis*. Mycoses 54, e816–e820. 10.1111/j.1439-0507.2011.02035.x 21672047

[B86] Favero D. Furlaneto-Maia L. Franca E. J. Goes H. P. Furlaneto M. C. (2014). Hemolytic factor production by clinical isolates of *Candida* species. Curr. Microbiol. 68, 161–166. 10.1007/s00284-013-0459-6 24048697

[B87] Fidel P. L. Cutright J. L. Tait L. Sobel J. D. (1996). A murine model of *Candida glabrata* vaginitis. J. Infect. Dis. 173, 425–431. 10.1093/infdis/173.2.425 8568305

[B88] Fidel P. Cutright J. L. Sobel J. D. (1997). Efficacy of D0870 treatment of experimental *Candida* vaginitis. Antimicrob. Agents Chemother. 41, 1455–1459. 9210665 10.1128/aac.41.7.1455 PMC163939

[B89] Figueiredo V. T. de Assis Santos D. Resende M. A. Hamdan J. S. (2007). Identification and *in vitro* antifungal susceptibility testing of 200 clinical isolates of *Candida* spp. responsible for fingernail infections. Mycopathologia 164, 27–33. 10.1007/s11046-007-9027-6 17551848

[B90] Finkel J. S. Mitchell A. P. (2011). Genetic control of *Candida albicans* biofilm development. Nat. Rev. Microbiol. 9, 109–118. 10.1038/nrmicro2475 21189476 PMC3891587

[B91] Fitzpatrick D. A. O'Gaora P. Byrne K. P. Butler G. (2010). Analysis of gene evolution and metabolic pathways using the *Candida* Gene Order Browser. BMC Genomics 11:290. 10.1186/1471-2164-11-290 20459735 PMC2880306

[B92] Fong P. H. Chan H. L. Lee Y. S. Wong H. B. (1988). Acute disseminated cutaneous candidiasis. Ann. Acad. Med. Singap. 17, 551–553. 3223743

[B93] Forastiero A. Mesa-Arango A. C. Alastruey-Izquierdo A. Alcazar-Fuoli L. Bernal-Martinez L. Pelaez T.. (2013). *Candida tropicalis* antifungal cross-resistance is related to different azole target (Erg11p) modifications. Antimicrob. Agents Chemother. 57, 4769–4781. 10.1128/AAC.00477-13 23877676 PMC3811422

[B94] Fuchs B. B. Li Y. Li D. Johnston T. Hendricks G. Li G. (2016). Micafungin elicits an immunomodulatory effect in *Galleria mellonella* and mice. Mycopathologia 181, 17–25. 10.1007/s11046-015-9940-z 26384671 PMC4676791

[B95] Galan-Ladero M. A. Blanco-Blanco M. T. Hurtado C. Perez-Giraldo C. Blanco M. T. Gomez-Garcia A. C. (2013). Determination of biofilm production by *Candida tropicalis* isolated from hospitalized patients and its relation to cellular surface hydrophobicity, plastic adherence and filamentation ability. Yeast 30, 331–339. 10.1002/yea.2965 23775541

[B96] Galan-Ladero M. Blanco M. Sacristán B. Fernández-Calderón M. Pérez-Giraldo C. Gomez-Garcia A. (2010). Enzymatic activities of *Candida tropicalis* isolated from hospitalized patients. Med. Mycol. 48, 207–210. 10.3109/13693780902801242 19274599

[B97] Garcia M. J. Rios G. Ali R. Bellés J. M. Serrano R. (1997). Comparative physiology of salt tolerance in *Candida tropicalis* and *Saccharomyces cerevisiae*. Microbiology 143, 1125–1131. 10.1099/00221287-143-4-1125 9141675

[B98] Garcia-Effron G. Chua D. J. Tomada J. R. DiPersio J. Perlin D. S. Ghannoum M.. (2010). Novel FKS mutations associated with echinocandin resistance in *Candida* species. Antimicrob. Agents Chemother. 54, 2225–2227. 10.1128/AAC.00998-09 20145084 PMC2863628

[B99] Garcia-Effron G. Kontoyiannis D. P. Lewis R. E. Perlin D. S. (2008). Caspofungin-resistant *Candida tropicalis* strains causing breakthrough fungemia in patients at high risk for hematologic malignancies. Antimicrob. Agents Chemother. 52, 4181–4183. 10.1128/AAC.00802-08 18794386 PMC2573122

[B100] Garczewska B. Jarzynka S. Kus J. Skorupa W. Augustynowicz-Kopec E. (2016). Fungal infection of cystic fibrosis patients—single center experience. Pneumonol. Alergol. Pol. 84, 151–159. 10.5603/PiAP.2016.0017 27238177

[B101] Garvey E. Hoekstra W. Schotzinger R. Sobel J. Lilly E. Fidel P. (2015). Efficacy of the clinical agent VT-1161 against fluconazole-sensitive and-resistant *Candida albicans* in a murine model of vaginal candidiasis. Antimicrob. Agents Chemother. 59, 5567–5573. 10.1128/AAC.00185-15 26124165 PMC4538529

[B102] Ghannoum M. A. (2000). Potential role of phospholipases in virulence and fungal pathogenesis. Clin. Microbiol. Rev. 13, 122–143. 10.1128/CMR.13.1.122-143.2000 10627494 PMC88936

[B103] Giolo M. P. Svidzinski T. I. E. (2010). Fisiopatogenia, epidemiologia e diagnóstico laboratorial da candidemia. J. Bras. Patol. Med. Lab. 46, 225–234. 10.1590/S1676-24442010000300009

[B104] Giri S. Kindo A. J. (2012). A review of *Candida* species causing blood stream infection. Indian J. Med. Microbiol. 30, 270–278. 10.4103/0255-0857.99484 22885191

[B105] Gonzalez-Novo A. Correa-Bordes J. Labrador L. Sanchez M. Vazquez de Aldana C. R. Jimenez J. (2008). Sep7 is essential to modify septin ring dynamics and inhibit cell separation during *Candida albicans* hyphal growth. Mol. Biol. Cell. 19, 1509–1518. 10.1091/mbc.E07-09-0876 18234840 PMC2291409

[B106] Gorton R. L. Ramnarain P. Barker K. Stone N. Rattenbury S. McHugh T. D.. (2014). Comparative analysis of Gram's stain, PNA-FISH and Sepsityper with MALDI-TOF MS for the identification of yeast direct from positive blood cultures. Mycoses 57, 592–601. 10.1111/myc.12205 24862948

[B107] Grant M. L. Parajuli S. Deleon-Gonsalves R. Potula R. Truant A. L. (2016). Comparative evaluation of the BD phoenix yeast ID panel and remel RapID yeast plus system for yeast identification. Can. J. Infect. Dis. Med. Microbiol. 2016:4094932. 10.1155/2016/4094932 27366167 PMC4904582

[B108] Guinea J. Zaragoza O. Escribano P. Martin-Mazuelos E. Peman J. Sanchez-Reus F.. (2014). Molecular identification and antifungal susceptibility of yeast isolates causing fungemia collected in a population-based study in Spain in 2010 and 2011. Antimicrob. Agents Chemother. 58, 1529–1537. 10.1128/AAC.02155-13 24366741 PMC3957835

[B109] Gündeş S. Gulenc S. Bingol R. (2001). Comparative performance of Fungichrom I, Candifast and API 20C Aux systems in the identification of clinically significant yeasts. J. Med. Microbiol. 50, 1105–1110. 10.1099/0022-1317-50-12-1105 11761197

[B110] Gustin M. C. Albertyn J. Alexander M. Davenport K. (1998). MAP kinase pathways in the yeast Saccharomyces cerevisiae. Microbiol. Mol. Biol. Rev. 62, 1264–1300. 9841672 10.1128/mmbr.62.4.1264-1300.1998 PMC98946

[B111] Hall L. Le Febre K. M. Deml S. M. Wohlfiel S. L. Wengenack N. L. (2012). Evaluation of the yeast traffic light PNA FISH probes for identification of *Candida* species from positive blood cultures. J. Clin. Microbiol. 50, 1446–1448. 10.1128/JCM.06148-11 22238445 PMC3318520

[B112] Hamamoto H. Kurokawa K. Kaito C. Kamura K. Razanajatovo I. M. Kusuhara H.. (2004). Quantitative evaluation of the therapeutic effects of antibiotics using silkworms infected with human pathogenic microorganisms. Antimicrob. Agents Chemother. 48, 774–779. 10.1128/AAC.48.3.774-779.2004 14982763 PMC353159

[B113] Hawser S. P. Douglas L. J. (1995). Resistance of *Candida albicans* biofilms to antifungal agents *in vitro*. Antimicrob. Agents Chemother. 39, 2128–2131. 10.1128/AAC.39.9.2128 8540729 PMC162894

[B114] Hoover C. I. Jantapour M. J. Newport G. Agabian N. Fisher S. J. (1998). Cloning and regulated expression of the *Candida albicans* phospholipase B (PLB1) gene. FEMS Microbiol. Lett. 167, 163–169. 10.1111/j.1574-6968.1998.tb13223.x 9809417

[B115] Hoyer L. L. Fundyga R. Hecht J. E. Kapteyn J. C. Klis F. M. Arnold J. (2001). Characterization of agglutinin-like sequence genes from non-*albicans Candida* and phylogenetic analysis of the ALS family. Genetics 157, 1555–1567. 11290712 10.1093/genetics/157.4.1555 PMC1461614

[B116] Hube B. Naglik J. (2001). *Candida albicans* proteinases: resolving the mystery of a gene family. Microbiology 147(Pt 8), 1997–2005. 10.1099/00221287-147-8-1997 11495978

[B117] Ishii M. Matsumoto Y. Sekimizu K. (2015). Usefulness of silkworm as a model animal for understanding the molecular mechanisms of fungal pathogenicity. Drug Discov. Ther. 9, 234–237. 10.5582/ddt.2015.01052 26370522

[B118] Jacobs P. H. Nall L. (1990). Antifungal Drug Therapy: A Complete Guide for the Practitioner. New York, NY: CRC Press.

[B119] Jain M. Shah R. Chandolia B. Mathur A. Chauhan Y. Chawda J. (2016). The oral carriage of *Candida* in oral cancer patients of indian origin undergoing radiotherapy and/or chemotherapy. J. Clin. Diagn. Res. 10, ZC17–ZC20. 10.7860/JCDR/2016/15702.7180 PMC4800644 27042578

[B120] Jensen R. H. Johansen H. K. Arendrup M. C. (2013). Stepwise development of a homozygous S80P substitution in Fks1p, conferring echinocandin resistance in *Candida tropicalis*. Antimicrob. Agents Chemother. 57, 614–617. 10.1128/AAC.01193-12 23089761 PMC3535961

[B121] Jiang C. Dong D. Yu B. Cai G. Wang X. Ji Y.. (2013). Mechanisms of azole resistance in 52 clinical isolates of *Candida tropicalis* in China. J. Antimicrob. Chemother. 68, 778–785. 10.1093/jac/dks481 23221625

[B122] Jiang C. Li Z. Zhang L. Tian Y. Dong D. Peng Y. (2016). Significance of hyphae formation in virulence of *Candida tropicalis* and transcriptomic analysis of hyphal cells. Microbiol. Res. 192, 65–72. 10.1016/j.micres.2016.06.003 27664724

[B123] Joseph-Horne T. Hollomon D. W. (1997). Molecular mechanisms of azole resistance in fungi. FEMS Microbiol. Lett. 149, 141–149. 10.1111/j.1574-6968.1997.tb10321.x 9141655

[B124] Kali A. Charles M. P. Noyal M. J. Sivaraman U. Kumar S. Easow J. M. (2013). Prevalence of *Candida* co-infection in patients with pulmonary tuberculosis. Australas. Med. J. 6, 387–391. 10.4066/AMJ.2013.1709 24039631 PMC3767025

[B125] Kanagal D. Vineeth V. Kundapur R. Shetty H. Rajesh A. (2014). Prevalence of vaginal candidiasis in pregnancy among coastal south Indian women. J. Womens Health. Issues Care 3:6 10.4172/2325-9795.1000168

[B126] Keceli S. A. Dundar D. Tamer G. S. (2016). Comparison of vitek matrix-assisted laser desorption/ionization time-of-flight mass spectrometry versus conventional methods in *Candida* identification. Mycopathologia 181, 67–73. 10.1007/s11046-015-9944-8 26400863

[B127] Kelly S. L. Arnoldi A. Kelly D. E. (1993). Molecular genetic analysis of azole antifungal mode of action. Biochem. Soc. Trans. 21, 1034–1038. 10.1042/bst0211034 8131893

[B128] Kim D. M. Suh M. K. Ha G. Y. (2013). Onychomycosis in children: an experience of 59 cases. Ann. Dermatol. 25, 327–334. 10.5021/ad.2013.25.3.327 24003276 PMC3756198

[B129] Kirk P. Cannon P. David J. Stalpers J. (2001). Ainsworth & Bisby's Dicitionary of the Fungi. Wallingford: Ed CAB International.

[B130] Klotz S. A. Drutz D. J. Harrison J. L. Huppert M. (1983). Adherence and penetration of vascular endothelium by *Candida* yeasts. Infect. Immun. 42, 374–384. 6352500 10.1128/iai.42.1.374-384.1983 PMC264568

[B131] Koga-Ito C. Y. Komiyama E. Y. de Paiva Martins C. A. Vasconcellos T. C. Cardoso Jorge A. O. Carvalho Y. R.. (2011). Experimental systemic virulence of oral *Candida dubliniensis* isolates in comparison with *Candida albicans, Candida tropicalis* and *Candida krusei*. Mycoses 54, e278–e85. 10.1111/j.1439-0507.2010.01899.x 20492535

[B132] Kontoyiannis D. P. Vaziri I. Hanna H. A. Boktour M. Thornby J. Hachem R.. (2001). Risk factors for *Candida tropicalis* fungemia in patients with cancer. Clin. Infect. Dis. 33, 1676–1681. 10.1086/323812 11568858

[B133] Kothavade R. J. Kura M. M. Valand A. G. Panthaki M. H. (2010). *Candida tropicalis*: its prevalence, pathogenicity and increasing resistance to fluconazole. J. Med. Microbiol. 59(Pt 8), 873–880. 10.1099/jmm.0.013227-0 20413622

[B134] Krauke Y. Sychrova H. (2008). Functional comparison of plasma-membrane Na^+^/H^+^ antiporters from two pathogenic *Candida* species. BMC Microbiol. 8:80. 10.1186/1471-2180-8-80 18492255 PMC2424070

[B135] Krcmery V. Jr. Mrazova M. Kunova A. Grey E. Mardiak J. Jurga L.. (1999). Nosocomial Candidaemias due to species other than *Candida albicans* in cancer patients. Support. Care Cancer 7, 428–431. 10.1007/s005200050304 10541986

[B136] Kühn C. Klipp E. (2012). Zooming in on yeast osmoadaptation. Adv. Sys. Biol. 739, 293–310. 10.1007/978-1-4419-7210-1_17 22161336

[B137] Kumamoto C. A. Vinces M. D. (2005). Contributions of hyphae and hypha-co-regulated genes to *Candida albicans* virulence. Cell. Microbiol. 7, 1546–1554. 10.1111/j.1462-5822.2005.00616.x 16207242

[B138] Kurtzman C. Fell J. W. Boekhout T. (2011). The Yeasts: A Taxonomic Study. Amsterdam: Elsevier.

[B139] Kwon S. G. Park S. W. Oh D. K. (2006). Increase of xylitol productivity by cell-recycle fermentation of *Candida tropicalis* using submerged membrane bioreactor. J. Biosci. Bioeng. 101, 13–18. 10.1263/jbb.101.13 16503285

[B140] Lackey E. Vipulanandan G. Childers D. S. Kadosh D. (2013). Comparative evolution of morphological regulatory functions in *Candida* species. Eukaryot. Cell 12, 1356–1368. 10.1128/EC.00164-13 23913541 PMC3811340

[B141] Legrand M. Forche A. Selmecki A. Chan C. Kirkpatrick D. T. Berman J. (2008). Haplotype mapping of a diploid non-meiotic organism using existing and induced aneuploidies. PLoS Genet. 4:e1. 10.1371/journal.pgen.0040001 18179283 PMC2174976

[B142] Leitão A. L. Duarte M. P. Oliveira J. S. (2007). Degradation of phenol by a halotolerant strain of *Penicillium chrysogenum*. Int. Biodeterior. Biodegrad. 59, 220–225. 10.1016/j.ibiod.2006.09.009

[B143] Li L. Zhang C. Konopka J. B. (2012). A *Candida albicans* temperature-sensitive *cdc12-6* mutant identifies roles for septins in selection of sites of germ tube formation and hyphal morphogenesis. Eukaryot. Cell 11, 1210–1218. 10.1128/EC.00216-12 22886998 PMC3485918

[B144] Liu W. Tan J. Sun J. Xu Z. Li M. Yang Q.. (2014). Invasive candidiasis in intensive care units in China: *in vitro* antifungal susceptibility in the China-SCAN study. J. Antimicrob. Chemother. 69, 162–167. 10.1093/jac/dkt330 24004860

[B145] Luo G. Samaranayake L. P. Yau J. Y. (2001). *Candida* species exhibit differential *in vitro* hemolytic activities. J. Clin. Microbiol. 39, 2971–2974. 10.1128/JCM.39.8.2971-2974.2001 11474025 PMC88272

[B146] Luo G. Samaranayake L. P. Cheung B. P. Tang G. (2004). Reverse transcriptase polymerase chain reaction (RT-PCR) detection of HLP gene expression in *Candida* glabrata and its possible role in *in vitro* haemolysin production. APMIS 112, 283–290. 10.1111/j.1600-0463.2004.apm11204-0509.x 15233644

[B147] Lupetti A. Danesi R. Campa M. Del Tacca M. Kelly S. (2002). Molecular basis of resistance to azole antifungals. Trends Mol. Med. 8, 76–81. 10.1016/S1471-4914(02)02280-3 11815273

[B148] Lyon J. P. de Resende M. A. (2006). Correlation between adhesion, enzyme production, and susceptibility to fluconazole in *Candida albicans* obtained from denture wearers. Oral Surg. Oral Med. Oral Pathol. Oral Radiol. Endod. 102, 632–638. 10.1016/j.tripleo.2005.12.015 17052640

[B149] Macdonald F. Odds F. C. (1983). Virulence for mice of a proteinase-secreting strain of *Candida albicans* and a proteinase-deficient mutant. J. Gen. Microbiol. 129, 431–438. 10.1099/00221287-129-2-431 6341508

[B150] Maiden M. C. Bygraves J. A. Feil E. Morelli G. Russell J. E. Urwin R.. (1998). Multilocus sequence typing: a portable approach to the identification of clones within populations of pathogenic microorganisms. Proc. Natl. Acad. Sci. U.S.A. 95, 3140–3145. 10.1073/pnas.95.6.3140 9501229 PMC19708

[B151] Manastir L. Ergon M. C. Yucesoy M. (2011). Investigation of mutations in Erg11 gene of fluconazole resistant *Candida albicans* isolates from Turkish hospitals. Mycoses 54, 99–104. 10.1111/j.1439-0507.2009.01766.x 19732347

[B152] Mancera E. Porman A. M. Cuomo C. A. Bennett R. J. Johnson A. D. (2015). Finding a missing gene: EFG1 regulates morphogenesis in *Candida tropicalis*. G3 (Bethesda). 5, 849–856. 10.1534/g3.115.017566 25758825 PMC4426371

[B153] Manns J. M. Mosser D. M. Buckley H. R. (1994). Production of a hemolytic factor by *Candida albicans*. Infect. Immun. 62, 5154–5156. 7927798 10.1128/iai.62.11.5154-5156.1994 PMC303238

[B154] Manzano-Gayosso P. Mendez-Tovar L. J. Arenas R. Hernandez-Hernandez F. Millan-Chiu B. Torres-Rodriguez J. M.. (2011). Onychomycosis-causing yeasts in four Mexican dermatology centers and their antifungal susceptibility to azolic compounds. Rev. Iberoam. Micol. 28, 32–35. 10.1016/j.riam.2010.11.002 21147249

[B155] Marcos J. Y. Pincus D. H. (2013). Fungal diagnostics: review of commercially available methods. Methods Mol. Biol. 968, 25–54. 10.1007/978-1-62703-257-5_2 23296883

[B156] Marcos-Zambrano L. J. Escribano P. Bouza E. Guinea J. (2014). Production of biofilm by *Candida* and non-*Candida* spp. isolates causing fungemia: comparison of biomass production and metabolic activity and development of cut-off points. Int. J. Med. Microbiol. 304, 1192–1198. 10.1016/j.ijmm.2014.08.012 25224357

[B157] Marie C. White T. C. (2009). Genetic basis of antifungal drug resistance. Curr. Fungal Infect. Rep. 3, 163–169. 10.1007/s12281-009-0021-y 20161440 PMC2790137

[B158] Mariné M. Pastor F. J. Guarro J. (2010). Efficacy of posaconazole in a murine disseminated infection by *Candida tropicalis*. Antimicrob. Agents Chemother. 54, 530–532. 10.1128/AAC.01136-09 19841151 PMC2798508

[B159] Marol S. Yücesoy M. (2008). Molecular epidemiology of *Candida* species isolated from clinical specimens of intensive care unit patients. Mycoses 51, 40–49. 10.1111/j.1439-0507.2007.01435.x 18076594

[B160] McCarty T. P. Pappas P. G. (2016). Invasive candidiasis. Infect. Dis. Clin. North Am. 30, 103–124. 10.1016/j.idc.2015.10.013 26739610

[B161] Melo A. S. Colombo A. L. Arthington-Skaggs B. A. (2007). Paradoxical growth effect of caspofungin observed on biofilms and planktonic cells of five different *Candida* species. Antimicrob. Agents Chemother. 51, 3081–3088. 10.1128/AAC.00676-07 17591847 PMC2043224

[B162] Mendes de Toledo C. E. M. Santos P. R. de Mello J. C. P. Filho B. P. D. Nakamura C. V. Ueda-Nakamura T. (2015). Antifungal properties of crude extracts, fractions, and purified compounds from bark of *Curatella americana* L. (Dilleniaceae) against *Candida* species. Evid. Based Complement. Alternat. Med. 2015:673962. 10.1155/2015/673962 26347790 PMC4548135

[B163] Menezes T. O. Gillet L. C. Menezes S. A. Feitosa R. N. Ishak M. O. Ishak R.. (2013). Virulence factors of *Candida albicans* isolates from the oral cavities of HIV-1-positive patients. Curr. HIV Res. 11, 304–308. 10.2174/1570162X113119990042 23822800

[B164] Merseguel K. B. Nishikaku A. S. Rodrigues A. M. Padovan A. C. Ferreira R. C. de Azevedo Melo A. S.. (2015). Genetic diversity of medically important and emerging *Candida* species causing invasive infection. BMC Infect. Dis. 15:57. 10.1186/s12879-015-0793-3 25887032 PMC4339437

[B165] Merz W. G. Sandford G. R. (1979). Isolation and characterization of a polyene-resistant variant of *Candida tropicalis*. J. Clin. Microbiol. 9, 677–680. 387815 10.1128/jcm.9.6.677-680.1979 PMC275378

[B166] Mesa-Arango A. C. Forastiero A. Bernal-Martínez L. Cuenca-Estrella M. Mellado E. Zaragoza O. (2013). The non-mammalian host Galleria mellonella can be used to study the virulence of the fungal pathogen *Candida tropicalis* and the efficacy of antifungal drugs during infection by this pathogenic yeast. Med. Mycol. 51, 461–472. 10.3109/13693786.2012.737031 23170962

[B167] Millsop J. W. Fazel N. (2016). Oral candidiasis. Clin. Dermatol. 34, 487–494. 10.1016/j.clindermatol.2016.02.022 27343964

[B168] Misra S. Raghuwanshi S. Gupta P. Dutt K. Saxena R. K. (2012). Fermentation behavior of osmophilic yeast *Candida tropicalis* isolated from the nectar of *Hibiscus rosa* sinensis flowers for xylitol production. Antonie Van Leeuwenhoek 101, 393–402. 10.1007/s10482-011-9646-2 21956659

[B169] Morais-Braga M. F. B. Sales D. L. Carneiro J. N. P. Machado A. J. T. dos Santos A. T. L. de Freitas M. A.. (2016). *Psidium guajava* L. *and Psidium brownianum M*art ex DC.: Chemical composition and anti–*Candida* effect in association with fluconazole. Microb. Pathog. 95, 200–207. 10.1016/j.micpath.2016.04.013 27085299

[B170] Morrow C. A. Fraser J. A. (2013). Ploidy variation as an adaptive mechanism in human pathogenic fungi. Semin. Cell Dev. Biol. 4, 339–346. 10.1016/j.semcdb.2013.01.008 23380396

[B171] Morschhäuser J. (2010). Regulation of multidrug resistance in pathogenic fungi. Fungal Genet. Biol. 47, 94–106. 10.1016/j.fgb.2009.08.002 19665571

[B172] Muadcheingka T. Tantivitayakul P. (2015). Distribution of *Candida albicans* and non-*albicans Candida* species in oral candidiasis patients: correlation between cell surface hydrophobicity and biofilm forming activities. Arch. Oral Biol. 60, 894–901. 10.1016/j.archoralbio.2015.03.002 25819801

[B173] Mulu A. Kassu A. Anagaw B. Moges B. Gelaw A. Alemayehu M.. (2013). Frequent detection of ‘azole’ resistant *Candida* species among late presenting AIDS patients in northwest Ethiopia. BMC Infect. Dis. 13:82. 10.1186/1471-2334-13-82 23398783 PMC3577436

[B174] Nagaraja P. Mathew T. Shetty D. (2005). *Candida tropicalis* causing prosthetic valve endocarditis. Indian J. Med. Microbiol. 23:139. 10.4103/0255-0857.16059 15928449

[B175] Naglik J. R. Fostira F. Ruprai J. Staab J. F. Challacombe S. J. Sundstrom P. (2006). *Candida albicans* HWP1 gene expression and host antibody responses in colonization and disease. J. Med. Microbiol. 55(Pt 10), 1323–1327. 10.1099/jmm.0.46737-0 17005778 PMC3244616

[B176] Nash E. E. Peters B. M. Fidel P. L. Noverr M. C. (2016a). Morphology-independent virulence of *Candida* species during polymicrobial intra-abdominal infections with Staphylococcus aureus. Infect. Immun. 84, 90–98. 10.1128/IAI.01059-15 26483410 PMC4694008

[B177] Nash E. E. Peters B. M. Lilly E. A. Noverr M. C. Fidel P. L. Jr. (2016b). A murine model of *Candida glabrata* Vaginitis shows no evidence of an inflammatory immunopathogenic response. PLoS ONE 11:e0147969. 10.1371/journal.pone.0147969 26807975 PMC4726552

[B178] Negri M. Martins M. Henriques M. Svidzinski T. I. Azeredo J. Oliveira R. (2010). Examination of potential virulence factors of *Candida tropicalis* clinical isolates from hospitalized patients. Mycopathologia 169, 175–182. 10.1007/s11046-009-9246-0 19851885

[B179] Nguyen V. Q. Sil A. (2008). Temperature-induced switch to the pathogenic yeast form of *Histoplasma capsulatum* requires Ryp1, a conserved transcriptional regulator. Proc. Natl. Acad. Sci. U.S.A. 105, 4880–4885. 10.1073/pnas.0710448105 18339808 PMC2290814

[B180] Nickerson K. W. Atkin A. L. Hornby J. M. (2006). Quorum sensing in dimorphic fungi: farnesol and beyond. Appl. Environ. Microbiol. 72, 3805–3813. 10.1128/AEM.02765-05 16751484 PMC1489610

[B181] Nobile C. J. Mitchell A. P. (2006). Genetics and genomics of *Candida albicans* biofilm formation. Cell. Microbiol. 8, 1382–1391. 10.1111/j.1462-5822.2006.00761.x 16848788

[B182] Nobile C. J. Schneider H. A. Nett J. E. Sheppard D. C. Filler S. G. Andes D. R.. (2008). Complementary adhesin function in C. *albicans* biofilm formation. Curr. Biol. 18, 1017–1024. 10.1016/j.cub.2008.06.034 18635358 PMC2504253

[B183] Nordin M. A. Wan Harun W. H. Abdul Razak F. (2013). Antifungal susceptibility and growth inhibitory response of oral *Candida* species to *Brucea javanica* Linn. extract. BMC Complement. Altern. Med. 13:342. 10.1186/1472-6882-13-342 24305010 PMC3898397

[B184] Nwibo D. D. Hamamoto H. Matsumoto Y. Kaito C. Sekimizu K. (2015). Current use of silkworm larvae (*Bombyx mori*) as an animal model in pharmaco-medical research. Drug Discov. Ther. 9, 133–135. 10.5582/ddt.2015.01026 25994065

[B185] Odds F. C. Jacobsen M. D. (2008). Multilocus sequence typing of pathogenic *Candida* species. Eukaryot. Cell 7, 1075–1084. 10.1128/EC.00062-08 18456859 PMC2446668

[B186] Okamoto-Shibayama K. Kikuchi Y. Kokubu E. Sato Y. Ishihara K. (2014). Csa2, a member of the Rbt5 protein family, is involved in the utilization of iron from human hemoglobin during *Candida albicans* hyphal growth. FEMS Yeast Res. 14, 674–677. 10.1111/1567-1364.12160 24796871

[B187] Oksuz S. Sahin I. Yildirim M. Gulcan A. Yavuz T. Kaya D.. (2007). Phospholipase and proteinase activities in different *Candida* species isolated from anatomically distinct sites of healthy adults. Jpn. J. Infect. Dis. 60, 280–283. 17881867

[B188] Oliveira V. K. P. D. (2011). Ocorrência das Espécies de Leveduras Isoladas de Sangue e Cateter de Pacientes Internados em Hospital Público Infantil de São Paulo (Período 2007 a 2010). Master's thesis, Universidade de São Paulo, São Paulo.

[B189] Paiva L. C. Vidigal P. G. Donatti L. Svidzinski T. I. Consolaro M. E. (2012). Assessment of *in vitro* biofilm formation by *Candida* species isolates from vulvovaginal candidiasis and ultrastructural characteristics. Micron 43, 497–502. 10.1016/j.micron.2011.09.013 22001373

[B190] Pam V. K. Akpan J. U. Oduyebo O. O. Nwaokorie F. O. Fowora M. A. Oladele R. O.. (2012). Fluconazole susceptibility and ERG11 gene expression in vaginal *Candida* species isolated from Lagos Nigeria. Int. J. Mol. Epidemiol. Genet. 3, 84–90. 22493755 PMC3316451

[B191] Panda A. Ghosh A. K. Mirdha B. R. Xess I. Paul S. Samantaray J. C.. (2015). MALDI-TOF mass spectrometry for rapid identification of clinical fungal isolates based on ribosomal protein biomarkers. J. Microbiol. Methods 109, 93–105. 10.1016/j.mimet.2014.12.014 25541362

[B192] Pannanusorn S. Fernandez V. Romling U. (2013). Prevalence of biofilm formation in clinical isolates of *Candida* species causing bloodstream infection. Mycoses 56, 264–272. 10.1111/myc.12014 23113805

[B193] Park S. Kelly R. Kahn J. N. Robles J. Hsu M. J. Register E.. (2005). Specific substitutions in the echinocandin target Fks1p account for reduced susceptibility of rare laboratory and clinical *Candida* sp. isolates. Antimicrob Agents Chemother. 49, 3264–3273. 10.1128/AAC.49.8.3264-3273.2005 16048935 PMC1196231

[B194] Peman J. Canton E. Quindos G. Eraso E. Alcoba J. Guinea J.. (2012). Epidemiology, species distribution and *in vitro* antifungal susceptibility of fungaemia in a Spanish multicentre prospective survey. J. Antimicrob. Chemother. 67, 1181–1187. 10.1093/jac/dks019 22351683

[B195] Perlin D. S. (2007). Resistance to echinocandin-class antifungal drugs. Drug Resist. Updat. 10, 121–130. 10.1016/j.drup.2007.04.002 17569573 PMC2696280

[B196] Pfaller M. A. (2012). Antifungal drug resistance: mechanisms, epidemiology, and consequences for treatment. Am. J. Med. 125(1 Suppl.), S3–S13. 10.1016/j.amjmed.2011.11.001 22196207

[B197] Pfaller M. A. Boyken L. Hollis R. J. Kroeger J. Messer S. A. Tendolkar S.. (2008). *In vitro* susceptibility of invasive isolates of *Candida* spp. to anidulafungin, caspofungin, and micafungin: six years of global surveillance. J Clin Microbiol. 46, 150–156. 10.1128/JCM.01901-07 18032613 PMC2224271

[B198] Pfaller M. A. Castanheira M. Diekema D. J. Messer S. A. Moet G. J. Jones R. N. (2010). Comparison of European Committee on Antimicrobial Susceptibility Testing (EUCAST) and Etest methods with the CLSI broth microdilution method for echinocandin susceptibility testing of *Candida* species. J. Clin. Microbiol. 48, 1592–1599. 10.1128/JCM.02445-09 20335424 PMC2863935

[B199] Pincus D. H. Orenga S. Chatellier S. (2007). Yeast identification–past, present, and future methods. Med. Mycol. 45, 97–121. 10.1080/13693780601059936 17365647

[B200] Porman A. M. Alby K. Hirakawa M. P. Bennett R. J. (2011). Discovery of a phenotypic switch regulating sexual mating in the opportunistic fungal pathogen *Candida tropicalis*. Proc. Natl. Acad. Sci. U.S.A. 108, 21158–21163. 10.1073/pnas.1112076109 22158989 PMC3248515

[B201] Porman A. M. Hirakawa M. P. Jones S. K. Wang N. Bennett R. J. (2013). MTL-independent phenotypic switching in *Candida tropicalis* and a dual role for Wor1 in regulating switching and filamentation. PLoS Genet. 9:e1003369. 10.1371/journal.pgen.1003369 23555286 PMC3605238

[B202] Posteraro B. Efremov L. Leoncini E. Amore R. Posteraro P. Ricciardi W.. (2015). Are the conventional commercial yeast identification methods still helpful in the era of new clinical microbiology diagnostics? A meta-analysis of their accuracy. J. Clin. Microbiol. 53, 2439–2450. 10.1128/JCM.00802-15 25994160 PMC4508456

[B203] Powderly W. G. Kobayashi G. S. Herzig G. P. Medoff G. (1988). Amphotericin B-resistant yeast infection in severely immunocompromised patients. Am. J. Med. 84, 826–832. 10.1016/0002-9343(88)90059-9 3284339

[B204] Punithavathy P. Menon T. (2012). Characterization of gene family that mediates the adhesion of biofilms formed by *Candida tropicalis* isolated from HIV and non-HIV patients. BMC Infect. Dis. 12:1 10.1186/1471-2334-12-S1-O822214291

[B205] Ra C. H. Jung J. H. Sunwoo I. Y. Kang C. H. Jeong G. T. Kim S. K. (2015). Detoxification of *Eucheuma spinosum* hydrolysates with activated carbon for ethanol production by the salt-tolerant yeast *Candida tropicalis*. J. Microbiol. Biotechnol. 25, 856–862. 10.4014/jmb.1409.09038 25649983

[B206] Ragunathan L. Poongothai G. K. Sinazer A. R. Kannaiyan K. Gurumurthy H. Jaget N.. (2014). Phenotypic characterization and antifungal susceptibility pattern to fluconazole in *Candida* species isolated from vulvovaginal candidiasis in a tertiary care hospital. J. Clin. Diagn. Res. 8, DC01–DC04. 10.7860/JCDR/2014/7434.4311 24995172 PMC4079993

[B207] Ramage G. Martinez J. P. Lopez-Ribot J. L. (2006). *Candida* biofilms on implanted biomaterials: a clinically significant problem. FEMS Yeast Res. 6, 979–986. 10.1111/j.1567-1364.2006.00117.x 17042747

[B208] Ramage G. Vande Walle K. Wickes B. L. Lopez-Ribot J. L. (2001). Standardized method for *in vitro* antifungal susceptibility testing of *Candida albicans* biofilms. Antimicrob. Agents Chemother. 45, 2475–2479. 10.1128/AAC.45.9.2475-2479.2001 11502517 PMC90680

[B209] Rao R. S. Jyothi C. P. Prakasham R. S. Sarma P. N. Rao L. V. (2006). Xylitol production from corn fiber and sugarcane bagasse hydrolysates by *Candida tropicalis*. Bioresour. Technol. 97, 1974–1978. 10.1016/j.biortech.2005.08.015 16242318

[B210] Rodriguez P. L. Ali R. Serrano R. (1996). CtCdc55p and CtHal3p: Two putative regulatory proteins from *Candida tropicalis* with long acidic domains. Yeast 12, 1321–1329. 10.1002/(SICI)1097-0061(199610)12:13<1321::AID-YEA27>3.0.CO;2-6 8923737

[B211] Rossoni R. D. Barbosa J. O. Vilela S. F. Jorge A. O. Junqueira J. C. (2013). Comparison of the hemolytic activity between *C. albicans* and non-albicans *Candida* species. Braz. Oral Res. 27, 484–489. 10.1590/S1806-83242013000600007 24346046

[B212] Ruchel R. Uhlemann K. Boning B. (1983). Secretion of acid proteinases by different species of the genus *Candida*. Zentralbl. Bakteriol. Mikrobiol. Hyg. A. 255, 537–548. 10.1016/S0174-3031(83)80013-4 6197827

[B213] Sachin C. Ruchi K. Santosh S. (2012). *In vitro* evaluation of proteinase, phospholipase and haemolysin activities of *Candida* species isolated from clinical specimens. IJMBR 1, 153–157. 10.14194/ijmbr.1211

[B214] Salari S. Bakhshi T. Sharififar F. Naseri A. Ghasemi N. A. P. (2016). Evaluation of antifungal activity of standardized extract of *Salvia rhytidea* Benth. *(*Lamiaceae) against various *Candida* isolates. J. Mycol. Med. 26, 323–330. 10.1016/j.mycmed.2016.06.003 27499461

[B215] Salehei Z. Seifi Z. Mahmoudabadi A. (2012). Sensitivity of vaginal isolates of *Candida* to eight antifungal drugs isolated from Ahvaz, Iran. Jundishapur J. Microbiol. 5, 574–577. 10.5812/jjm.4556

[B216] Samaranayake L. P. Raeside J. M. MacFarlane T. W. (1984). Factors affecting the phospholipase activity of *Candida* species *in vitro*. Sabouraudia 22, 201–207. 6379916

[B217] Sanglard D. Odds F. C. (2002). Resistance of *Candida* species to antifungal agents: molecular mechanisms and clinical consequences. Lancet Infect. Dis. 2, 73–85. 10.1016/S1473-3099(02)00181-0 11901654

[B218] Sanita P. V. Zago C. E. Mima E. G. Pavarina A. C. Jorge J. H. Machado A. L. (2014). *In vitro* evaluation of the enzymatic activity profile of non-*albicans Candida* species isolated from patients with oral candidiasis with or without diabetes. Oral Surg. Oral Med. Oral Pathol. Oral Radiol. 118, 84–91. 10.1016/j.oooo.2014.03.020 24908598

[B219] Santhanam J. Nazmiah N. Aziz M. N. (2013). Species distribution and antifungal susceptibility patterns of *Candida* species: is low susceptibility to itraconazole a trend in Malaysia? Med. J. Malaysia 68, 343–347. 24145264

[B220] Santos C. Lima N. Sampaio P. Pais C. (2011). Matrix-assisted laser desorption/ionization time-of-flight intact cell mass spectrometry to detect emerging pathogenic *Candida* species. Diagn. Microbiol. Infect. Dis. 71, 304–308. 10.1016/j.diagmicrobio.2011.07.002 21855250

[B221] Sariguzel F. Berk E. Koc A. Sav H. Aydemir G. (2015). Evaluation of CHROMagar *Candida*, VITEK2 YST and VITEK® MS for identification of *Candida* strains isolated from blood cultures. Infez. Med. 23, 318–322. 26700081

[B222] Schaller M. Borelli C. Korting H. C. Hube B. (2005). Hydrolytic enzymes as virulence factors of *Candida albicans*. Mycoses 48, 365–377. 10.1111/j.1439-0507.2005.01165.x 16262871

[B223] Seervai R. N. H. Jones S. K. Jr. Hirakawa M. P. Porman A. M. Bennett R. J. (2013). Parasexuality and ploidy change in *Candida tropicalis*. Eukaryot. Cell 12, 1629–1640. 10.1128/EC.00128-13 24123269 PMC3889571

[B224] Seneviratne C. J. Rajan S. Wong S. S. Tsang D. N. Lai C. K. Samaranayake L. P.. (2016). Antifungal susceptibility in serum and virulence determinants of *Candida* bloodstream isolates from Hong Kong. Front. Microbiol. 7:216. 10.3389/fmicb.2016.00216 26955369 PMC4767892

[B225] Shi X. Y. Yang Y. P. Zhang Y. Li W. Wang J. D. Huang W. M.. (2015). Molecular identification and antifungal susceptibility of 186 *Candida* isolates from vulvovaginal candidiasis in southern China. J. Med. Microbiol. 64, 390–393. 10.1099/jmm.0.000024 25596116

[B226] Shu C. Sun L. Zhang W. (2016). Thymol has antifungal activity against *Candida albicans* during infection and maintains the innate immune response required for function of the p38 MAPK signaling pathway in *Caenorhabditis elegans*. Immunol. Res. 64, 1013–1024. 10.1007/s12026-016-8785-y 26783030

[B227] Silva S. Hooper S. J. Henriques M. Oliveira R. Azeredo J. Williams D. W. (2011). The role of secreted aspartyl proteinases in *Candida tropicalis* invasion and damage of oral mucosa. Clin. Microbiol. Infect. 17, 264–722. 10.1111/j.1469-0691.2010.03248.x 20456460

[B228] Silva S. Negri M. Henriques M. Oliveira R. Williams D. W. Azeredo J. (2012). *Candida* glabrata, *Candida parapsilosis* and *Candida tropicalis*: biology, epidemiology, pathogenicity and antifungal resistance. FEMS Microbiol. Rev. 36, 288–305. 10.1111/j.1574-6976.2011.00278.x 21569057

[B229] Silva-Dias A. Miranda I. M. Rocha R. Monteiro-Soares M. Salvador A. Rodrigues A. G.. (2012). A novel flow cytometric protocol for assessment of yeast cell adhesion. Cytometry A 81, 265–270. 10.1002/cyto.a.21170 22076919

[B230] Siqueira A. B. S. Rodriguez L. R. N. D. A. Santos R. K. B. Marinho R. R. B. Abreu S. Peixoto R. F.. (2015). Antifungal activity of propolis against *Candida* species isolated from cases of chronic periodontitis. Braz. Oral Res. 29, 1–6. 10.1590/1807-3107BOR-2015.vol29.0083 26154370

[B231] Slutsky B. Staebell M. Anderson J. Risen L. Pfaller M. Soll D. (1987). White-opaque transition”: a second high-frequency switching system in *Candida albicans*. J. Bacteriol. 169, 189–197. 10.1128/jb.169.1.189-197.1987 3539914 PMC211752

[B232] Snide J. Sundstrom P. (2006). A characterization of HWP1 promoter activation in pseudohyphal cells in Candida albicans, in Proceedings of the 8th ASM Conference on Candida and Candidiasis (Denver, CO: ASM Press).

[B233] Sobel J. D. (2016). Recurrent vulvovaginal candidiasis. Am. J. Obstet. Gynecol. 214, 15–21. 10.1016/j.ajog.2015.06.067 26164695

[B234] Soczo G. Kardos G. Varga I. Kelentey B. Gesztelyi R. Majoros L. (2007). *In vitro* study of *Candida tropicalis* isolates exhibiting paradoxical growth in the presence of high concentrations of caspofungin. Antimicrob. Agents Chemother. 51, 4474–4476. 10.1128/AAC.00880-07 17923496 PMC2168010

[B235] Sohn K. Senyurek I. Fertey J. Konigsdorfer A. Joffroy C. Hauser N.. (2006). An *in vitro* assay to study the transcriptional response during adherence of *Candida albicans* to different human epithelia. FEMS Yeast Res. 6, 1085–1093. 10.1111/j.1567-1364.2006.00130.x 17042758

[B236] Solis N. V. Filler S. G. (2012). Mouse model of oropharyngeal candidiasis. Nat. Protoc. 7, 637–642. 10.1038/nprot.2012.011 22402633 PMC3671943

[B237] Souza A. C. R. Fuchs B. B. Pinhati H. M. Siqueira R. A. Hagen F. Meis J. F.. (2015). *Candida parapsilosis* resistance to fluconazole: molecular mechanisms and *in vivo* impact in infected *Galleria mellonella* larvae. Antimicrob. Agents Chemother. 59, 6581–6587. 10.1128/AAC.01177-15 26259795 PMC4576033

[B238] Sow D. Fall B. Ndiaye M. Ba B. S. Sylla K. Tine R.. (2015). Usefulness of MALDI-TOF mass spectrometry for routine identification of *Candida* species in a resource-poor setting. Mycopathologia 180, 173–179. 10.1007/s11046-015-9905-2 26016846

[B239] Stefaniuk E. Baraniak A. Fortuna M. Hryniewicz W. (2016). Usefulness of CHROMagar *Candida* medium, biochemical methods–API ID32C and VITEK 2 compact and two MALDI-TOF MS systems for *Candida* spp. identification. Pol. J. Microbiol. 65, 111–114. 10.5604/17331331.1197283 27282002

[B240] Stender H. (2003). PNA FISH: an intelligent stain for rapid diagnosis of infectious diseases. Expert Rev. Mol. Diagn. 3, 649–655. 10.1586/14737159.3.5.649 14510184

[B241] Stevens D. A. Espiritu M. Parmar R. (2004). Paradoxical effect of caspofungin: reduced activity against *Candida albicans* at high drug concentrations. Antimicrob. Agents Chemother. 48, 3407–3411. 10.1128/AAC.48.9.3407-3411.2004 15328104 PMC514730

[B242] Stone N. R. Gorton R. L. Barker K. Ramnarain P. Kibbler C. C. (2013). Evaluation of PNA-FISH yeast traffic light for rapid identification of yeast directly from positive blood cultures and assessment of clinical impact. J. Clin. Microbiol. 51, 1301–1302. 10.1128/JCM.00028-13 23390280 PMC3666762

[B243] Sun H. Y. Chiu Y. S. Tang J. L. Wang J. L. Chang S. C. Chen Y. C. (2006). The usefulness of the Platelia *Candida* antigen in a patient with acute lymphocytic leukemia and chronic disseminated candidiasis. Med. Mycol. 44, 647–650. 10.1080/13693780600735445 17071559

[B244] Sundstrom P. Balish E. Allen C. M. (2002). Essential role of the *Candida albicans* transglutaminase substrate, hyphal wall protein 1, in lethal oroesophageal candidiasis in immunodeficient mice. J. Infect. Dis. 185, 521–530. 10.1086/338836 11865405

[B245] Symersky J. Monod M. Foundling S. I. (1997). High-resolution structure of the extracellular aspartic proteinase from *Candida tropicalis* yeast. Biochemistry 36, 12700–12710. 10.1021/bi970613x 9335526

[B246] Takakura N. Sato Y. Ishibashi H. Oshima H. Uchida K. Yamaguchi H.. (2003). A novel murine model of oral candidiasis with local symptoms characteristic of oral thrush. Microbiol. Immun. 47, 321–326. 10.1111/j.1348-0421.2003.tb03403.x 12825893

[B247] Tavanti A. Davidson A. D. Johnson E. M. Maiden M. C. Shaw D. J. Gow N. A.. (2005). Multilocus sequence typing for differentiation of strains of *Candida tropicalis*. J. Clin. Microbiol. 43, 5593–5600. 10.1128/JCM.43.11.5593-5600.2005 16272492 PMC1287820

[B248] Ten Cate J. Klis F. Pereira-Cenci T. Crielaard W. De Groot P. (2009). Molecular and cellular mechanisms that lead to *Candida* biofilm formation. J. Dent. Res. 88, 105–115. 10.1177/0022034508329273 19278980

[B249] Togni G. Sanglard D. Falchetto R. Monod M. (1991). Isolation and nucleotide sequence of the extracellular acid protease gene (ACP) from the yeast *Candida tropicalis*. FEBS Lett. 286, 181–185. 10.1016/0014-5793(91)80969-A 1864366

[B250] Togni G. Sanglard D. Quadroni M. Foundling S. I. Monod M. (1996). Acid proteinase secreted by *Candida tropicalis*: functional analysis of preproregion cleavages in C. *tropicalis* and Saccharomyces cerevisiae. Microbiology 142 (Pt 3), 493–503. 10.1099/13500872-142-3-493 8868424

[B251] Tokuoka K. (1993). Sugar-and salt-tolerant yeasts. J. Appl. Microbiol. 74, 101–110. 10.1111/j.1365-2672.1993.tb03002.x

[B252] Torres M. P. Entwistle F. Coote P. J. (2016). Effective immunosuppression with dexamethasone phosphate in the *Galleria mellonella* larva infection model resulting in enhanced virulence of *Escherichia coli* and *Klebsiella pneumoniae*. Med. Microbiol. Immunol. 205, 333–343. 10.1007/s00430-016-0450-5 26920133 PMC4939170

[B253] Tronchin G. Pihet M. Lopes-Bezerra L. M. Bouchara J. P. (2008). Adherence mechanisms in human pathogenic fungi. Med. Mycol. 46, 749–772. 10.1080/13693780802206435 18651303

[B254] Tsang C. S. Chu F. C. Leung W. K. Jin L. J. Samaranayake L. P. Siu S. C. (2007). Phospholipase, proteinase and haemolytic activities of *Candida albicans* isolated from oral cavities of patients with type 2 diabetes mellitus. J. Med. Microbiol. 56(Pt 10), 1393–1398. 10.1099/jmm.0.47303-0 17893179

[B255] Uchida R. Namiguchi S. Ishijima H. Tomoda H. (2016). Therapeutic effects of three trichothecenes in the silkworm infection assay with *Candida albicans*. Drug Discov. Ther. 10, 44–48. 10.5582/ddt.2016.01013 26971555

[B256] Udayalaxmi Jacob S. D'Souza D. (2014). Comparison between virulence factors of *Candida albicans* and Non-Albicans species of *Candida* isolated from genitourinary tract. J Clin Diagn Res. 8, DC15–DC17. 10.7860/JCDR/2014/10121.5137 25584218 PMC4290236

[B257] Uppuluri P. Pierce C. G. Thomas D. P. Bubeck S. S. Saville S. P. Lopez-Ribot J. L. (2010). The transcriptional regulator Nrg1p controls *Candida albicans* biofilm formation and dispersion. Eukaryot. Cell 9, 1531–1537. 10.1128/EC.00111-10 20709787 PMC2950430

[B258] Vandeputte P. Tronchin G. Berges T. Hennequin C. Chabasse D. Bouchara J. P. (2007). Reduced susceptibility to polyenes associated with a missense mutation in the ERG6 gene in a clinical isolate of *Candida glabrata* with pseudohyphal growth. Antimicrob. Agents Chemother. 51, 982–9890. 10.1128/AAC.01510-06 17158937 PMC1803144

[B259] Verstrepen K. J. Klis F. M. (2006). Flocculation, adhesion and biofilm formation in yeasts. Mol. Microbiol. 60, 5–15. 10.1111/j.1365-2958.2006.05072.x 16556216

[B260] Vicari P. Feitosa Pinheiro R. de Chauffaille M. L. Yamamoto M. Figueiredo M. S. (2003). Septic arthritis as the first sign of *Candida tropicalis* fungaemia in an acute lymphoid leukemia patient. Braz. J. Infect. Dis. 7, 426–428. 10.1590/S1413-86702003000600012 14636484

[B261] Vijaya D. Dhanalakshmi T. A. Kulkarni S. (2014). Changing trends of vulvovaginal candidiasis. J. Lab. Phys. 6, 28–30. 10.4103/0974-2727.129087 24696557 PMC3969638

[B262] Vincent B. M. Lancaster A. K. Scherz-Shouval R. Whitesell L. Lindquist S. (2013). Fitness trade-offs restrict the evolution of resistance to amphotericin B. PLoS Biol. 11:e1001692. 10.1371/journal.pbio.1001692 24204207 PMC3812114

[B263] Wan Harun W. H. Jamil N. A. Jamaludin N. H. Nordin M. A. (2013). Effect of Piper betle and *Brucea javanica* on the differential expression of Hyphal Wall Protein (HWP1) in Non-*Candida albicans Candida* (NCAC) Species. Evid. Based Complement. Alternat. Med. 2013:397268. 10.1155/2013/397268 23853657 PMC3703345

[B264] Wang T. Pan D. Zhou Z. You Y. Jiang C. Zhao X.. (2016). Dectin-3 deficiency promotes colitis development due to impaired antifungal innate immune responses in the gut. PLoS Pathog. 12:e1005662. 10.1371/journal.ppat.1005662 27280399 PMC4900642

[B265] Wapinski I. Pfeffer A. Friedman N. Regev A. (2007). Natural history and evolutionary principles of gene duplication in fungi. Nature 449, 54–61. 10.1038/nature06107 17805289

[B266] Weber K. Schulz B. Ruhnke M. (2010). The quorum-sensing molecule E, E-farnesol—its variable secretion and its impact on the growth and metabolism of *Candida* species. Yeast 27, 727–739. 10.1002/yea.1769 20641010

[B267] Wei W.-j. Yang H.-f. Ye Y. Li J.-b. (2016). *Galleria mellonella* as a model system to assess the efficacy of antimicrobial agents against *Klebsiella pneumoniae* infection. J. Chemother. 29, 252–256. 10.1080/1120009X.2016.1156892 27237961

[B268] Won E. J. Shin J. H. Kim M. N. Choi M. J. Joo M. Y. Kee S. J.. (2014). Evaluation of the BD Phoenix system for identification of a wide spectrum of clinically important yeast species: a comparison with Vitek 2-YST. Diagn. Microbiol. Infect. Dis. 79, 477–480. 10.1016/j.diagmicrobio.2014.05.011 24952986

[B269] Woods R. A. Bard M. Jackson I. E. Drutz D. J. (1974). Resistance to polyene antibiotics and correlated sterol changes in two isolates of *Candida tropicalis* from a patient with an amphotericin B-resistant funguria. J. Infect. Dis. 129, 53–58. 10.1093/infdis/129.1.53 4587944

[B270] Wu Y. Zhou H. J. Che J. Li W. G. Bian F. N. Yu S. B.. (2014). Multilocus microsatellite markers for molecular typing of *Candida tropicalis* isolates. BMC Microbiol. 14:245. 10.1186/s12866-014-0245-z 25410579 PMC4247128

[B271] Wu Y. Zhou H. Wang J. Li L. Li W. Cui Z.. (2012). Analysis of the clonality of *Candida tropicalis* strains from a general hospital in Beijing using multilocus sequence typing. PLoS ONE 7:e47767. 10.1371/journal.pone.0047767 23152759 PMC3494695

[B272] Xie J. Du H. Guan G. Tong Y. Kourkoumpetis T. K. Zhang L.. (2012). N-acetylglucosamine induces white-to-opaque switching and mating in *Candida tropicalis*, providing new insights into adaptation and fungal sexual evolution. Eukaryot. Cell 11, 773–782. 10.1128/EC.00047-12 22544905 PMC3370467

[B273] Xu B. Shi P. Wu H. Guo X. Wang Q. Zhou S. (2010). Utility of FDG PET/CT in guiding antifungal therapy in acute leukemia patients with chronic disseminated candidiasis. Clin. Nucl. Med. 35, 567–750. 10.1097/RLU.0b013e3181e4db84 20631500

[B274] Yan J. Jianping W. Hongmei L. Suliang Y. Zongding H. (2005). The biodegradation of phenol at high initial concentration by the yeast *Candida tropicalis*. Biochem. Eng. J. 24, 243–247. 10.1016/j.bej.2005.02.016

[B275] Zanette R. A. Kontoyiannis D. P. (2013). Paradoxical effect to caspofungin in *Candida* species does not confer survival advantage in a Drosophila model of candidiasis. Virulence 4, 497–498. 10.4161/viru.25523 23863608 PMC5359725

[B276] Zaugg C. Borg-Von Zepelin M. Reichard U. Sanglard D. Monod M. (2001). Secreted aspartic proteinase family of *Candida tropicalis*. Infect. Immun. 69, 405–412. 10.1128/IAI.69.1.405-412.2001 11119531 PMC97897

[B277] Zhang Q. Tao L. Guan G. Yue H. Liang W. Cao C.. (2016). Regulation of filamentation in the human fungal pathogen *Candida tropicalis*. Mol. Microbiol. 99, 528–545. 10.1111/mmi.13247 26466925

[B278] Zhang Y. Tao L. Zhang Q. Guan G. Nobile C. J. Zheng Q.. (2016). The gray phenotype and tristable phenotypic transitions in the human fungal pathogen *Candida tropicalis*. Fungal Genet. Biol. 93, 10–16. 10.1016/j.fgb.2016.05.006 27246518

[B279] Zhao L. de Hoog G. S. Cornelissen A. Lyu Q. Mou L. Liu T.. (2016). Prospective evaluation of the chromogenic medium CandiSelect 4 for differentiation and presumptive identification of non-*Candida albicans Candida* species. Fungal Biol. 120, 173–178. 10.1016/j.funbio.2015.09.006 26781374

[B280] Zheng X. D. Lee R. T. Wang Y. M. Lin Q. S. Wang Y. (2007). Phosphorylation of Rga2, a Cdc42 GAP, by CDK/Hgc1 is crucial for *Candida albicans* hyphal growth. EMBO J. 26, 3760–3769. 10.1038/sj.emboj.7601814 17673907 PMC1952229

[B281] Zuza-Alves D. L. de Medeiros S. S. de Souza L. B. Silva-Rocha W. P. Francisco E. C. de Araújo M. C.. (2016). Evaluation of virulence factors *in vitro*, resistance to osmotic stress and antifungal susceptibility of *Candida tropicalis* isolated from the coastal environment of Northeast Brazil. Front. Microbiol. 7:1783. 10.3389/fmicb.2016.01783 27895625 PMC5108815

